# Nutrient-based diet modifications impact on the gut microbiome of the Javan slow loris (*Nycticebus javanicus*)

**DOI:** 10.1038/s41598-019-40911-0

**Published:** 2019-03-11

**Authors:** F. Cabana, J. B. Clayton, K. A. I. Nekaris, W. Wirdateti, D. Knights, H. Seedorf

**Affiliations:** 1 Wildlife Nutrition Centre, Wildlife Reserves Singapore, 80 Mandai Lake Road, 729826 Singapore, Singapore; 2 Nocturnal Primate Research Group, Oxford Brookes University, Gypsy Lane, Oxford, OX3 0BP UK; 3 Primate Microbiome Project, 6-124 MCB, 420 Washington Ave SE, Minneapolis, MN 55455 USA; 4 Department of Computer Science and Engineering, University of Minnesota, 4-192 Keller Hall, 200 Union St SE, Minneapolis, MN 55455 USA; 5 GreenViet Biodiversity Conservation Center, K39/21 Thanh Vinh Street, Son Tra District, Danang, Vietnam; 6 Biotechnology Institute, University of Minnesota, 1479 Gortner Avenue, Saint Paul, MN 55108 USA; 7 LIPI Zoology, Jalan Raya Bogor KM.46, Kel. Nanggewer Mekar, Kec. Cibinong, Cibinong, Bogor, Jawa Barat 16911 Indonesia; 8 Temasek Life Sciences Laboratory, 1 Research Link, Singapore, 117604 Singapore; 9 Department of Biological Sciences, National University of Singapore, Singapore, 117604 Singapore

## Abstract

Environment and diet are key factors which shape the microbiome of organisms. There is also a disparity between captive and wild animals of the same species, presumably because of the change in diet. Being able to reverse the microbiome to the wild type is thus particularly important for the reintroduction efforts of Critically Endangered animals. The Javan slow loris (*Nycticebus javanicus)* is a suitable model, being kept in the thousands within rescue centres throughout Southeast Asia. With next-generation sequencing, we show how a naturalistic diet impacts the gut microbiome of captive slow lorises (Primates: *Nycticebus*). A comparison of the microbiome of wild animals with captive animals that had been fed a standard captive or improved diet reveals strong microbiome differences between wild and captive animals; however, diet changes failed to alter the microbiome of captive populations significantly. *Bifidobacterium* was the most abundant genus in wild animals (46.7%) while *Bacteroides* (11.6%) and *Prevotella* (18.9%) were the most abundant in captive animals fed the captive and improved diets, respectively. Correlation analyses of nutrients with microbial taxa suggest important implications in using nutrition to suppress potential pathogens, with soluble fibre and water-soluble carbohydrates both being associated with opposing microbiome profiles. The improved diet significantly increased microbe diversity, which exemplifies the importance of high fibre diets; however, wild individuals had lower diversity, which contradicts recent studies. Detection of methanogens appeared to be dependent on diet and whether the animals were living in captivity or in the wild. This study highlights the potential of nutrition in modulating the microbiome of animals prior to release. Unexpectedly, the results were not as significant as has been suggested in recent studies.

## Introduction

Study of the feeding ecology of free-ranging animals has been conducted using a range of methods including proportion of feeding time^1^, faecal sampling^2^, ingestion rate^3^ and more recently metagenomics^4^ and faecal microbiome research^5,6^. Using empirical evidence to define the ecology of animals is essential to their conservation and the rebuilding of their habitat, as well as vital in identifying appropriate translocation areas^5^. Microbiome analyses have been used to categorise the gut microbial communities of a number of wild species, often with the aim of comparing them to congeners under human care^7^. Many health issues can now be linked with the microbiome, making this a promising area of research, especially regarding conservation translocations^8,9^. Gut microbe communities are affected by several variables, the largest of which may be genetics^10^ or diet^11–13^; therefore, as our understanding of the microbiome grows, it may become possible to predict the health state of an animal, especially when comparing with wild individuals of the same species.

Knowing how microbial communities respond to host health status could become a valuable tool in assessing which individuals are more suitable for translocation back into their natural habitat from a captive setting. Such information may be even more vital in the tropics, where rescue centres with limited budgets often provide inappropriate diets to some of the world’s most threatened species^14^. Assuming the microbiome of wild individuals is representative of a native state, environmental variables (including diet) must be tweaked to allow the host to maintain a wild-type microbiome. Gut microbial community structure has been variously shown to have an intimate link with immune system function^15^, parasite/pathogen colonisation^16,17^ and nutrient and energy assimilation^18,19^. A captive microbiome that is comparable to wild animals could therefore increase the success of translocation. Captivity may have a detrimental effect on animal microbiomes, as has been reported for primates, leading to a state of microbial imbalance otherwise known as dysbiosis^11,20^. Translocation is already an unpredictable and stressful event; releasing a primate with a dysbiotic microbiome may further reduce success rates, since their lowered microbial diversity may make them less adaptive to their environment and thus reduce their probability of survival^21^. Microbiomes can thus potentially impact an animal’s fitness.

A large body of work has already been done on non-human primates due to their use as models for human diseases, which allows for comparisons between species of similar digestive strategies and ecological niches^22^. Asia’s slow lorises (Primates, Lorisidae, *Nycticebus* spp.) are thus ideal models for studying the appropriateness of using microbiome research in assessing animals for release. All species of slow loris are listed on CITES Appendix 1, due to high demand for them in the illegal wildlife trade^23–25^. Because of this, thousands of slow lorises, many of which may be suitable for translocation^26^, are now found in rescue centres throughout Southeast Asia. Only a handful of systematic translocation studies are available^27–32^, with an average success rate of 10% that agrees with global translocation results^29,33^.

One of the most important differences observed thus far between captive and wild microbiomes appears to be the lower ratio of Firmicutes to Bacteroides in captive animals^11,22,34^. A higher ratio would suggest a more efficient capacity for energy harvesting as well as a larger production of beneficial short-chained fatty acids^19,35^. There is still much room for improvement on rehabilitation protocols of *Nycticebus* spp., including regarding nutrition. Rescue centres, typically restricted by budget, feed diets high in fruit and consequently high in soluble carbohydrates and low in fibre^26,36^. All slow loris species are characterised as exudativores, meaning they are specialised for ingesting tree gum made entirely of soluble fibres and other complex carbohydrates^37^. New evidence supports gum being a staple food item for slow lorises, rather than a fall-back food^36,38^. There may be a preference for Fabaeceae, followed by Anacardiaceae and Combretaceae trees; however, this may just be due to more trees within these families producing gum^38^.

The shift from a wild-type diet to a captive-type in the long term was predicted to lead to significant changes in their gut microbiome communities with possible deleterious health consequences^22,39^. A change in diet during the rehabilitation phase into one that resembles their wild diet may alter their microbiome and thus provide beneficial health effects. It may also be as a stepping stone leading to letting the primate fend for itself. The primary aim of this study was to compare the gut microbiomes of wild *Nycticebus* spp. with those of rescue-centre animals. We also looked at their microbiome composition on a typical sanctuary-fed diet (henceforth “typical diet”), and again after a diet change reflecting a nutrient intake closer to wild congeners (henceforth “improved diet”), to determine if the microbiome composition shifts to resemble the wild individuals more closely after a dietary change. Based on the current literature, we predict that the transition to an improved diet will shift their microbiome towards that of their wild congeners, specifically resulting in a higher Firmicutes to Bacteroides ratio. We also predict that the improved diet will lead to an increase in microbe diversity.

## Methods

### Slow loris sample collection

We collected ten faecal samples from two male and three female wild Javan slow lorises (*N*. *javanicus)* from Mt. Papandayan (S7°6′6″–7°7′ and E107°46′–107°46′5″) in West Java, Indonesia in May 2015. As part of a long-term study, we collared the slow lorises with radio collars (BioTrack, UK) and tracked them using a six-element Yagi antenna and SIKA receiver (BioTrack, UK)^40^. During their annual health checks, some individuals defecated during handling, directly into a plastic sample collection tube filled with 96% ethanol. After returning to base camp, we placed the vials in a freezer at −20 °C. We collected 23 captive faecal samples of *N*. *javanicus*, 11 of *N*. *coucang* and 3 of *N*. *menegensis* at Cikananga Wildlife Rescue Centre (CWRC - S7°03′27.04″ and E106°54′36.63″), West Java, Indonesia. Only faecal samples of healthy wild and captive individuals were used within the study. We define healthy as having no diagnosed health issue by the resident veterinarian. Variation in weight was allowed. We monitored captive individuals nightly using next generation LED headlamps with a red filter (CluLite, UK) and collected faecal samples from the concrete floor of their enclosures approximately ten minutes after defecation. We collected samples during their traditional diet treatment (fruits, insects and eggs), and two weeks after we transitioned to an improved diet to contain more wild food items (tree gum, insects, plant parts and nectar).

All methods and experimental protocols (both within the field and within the rescue centre) were performed in accordance with the relevant guidelines and regulations approved by Oxford Brookes University Ethics Board, as well as the Indonesian Research Institute (LIPI) in conjunction with the Kementerian Riset Teknologi Dan Pendidikan Tinggi (RISTEK) research permit (Foreign Research Permit # 163SPPRPBWUV2014).

### Wild and captive diet nutrient information

Wild ingested diets of *N*. *javanicus* comprised insects, tree gum and minor amounts of bamboo leaves, fruits and nectar^36^. This arrangement translates to a diet that contains on an average dry matter basis: 23.5% crude protein, 2.4% crude fat, 10.7% soluble fibre, 10.95% acid detergent fibre (ADF), 19.1% neutral detergent fibre (NDF), 0.45% calcium and 0.16% phosphorous, all on a dry matter basis^41^. Traditional captive diets (i.e. the typical diet) were composed of fruits, insects and eggs, giving an average nutrient content of 12.6% crude protein, 5.8% crude fat, 2.8% soluble fibre, 6.6% NDF, 0.23% calcium and 0.18% phosphorous, all on a dry matter basis. Wild diets had an average energy density of 3.63 kcal/g (15.19 kJ/g) and typical diets had an average energy concentration of 2.99 kcal/g (12.51 kJ/g). These values were significantly different from the nutrient concentration of the improved diet, made from vegetables, tree gum and insects. Slow lorises on the improved diet ingested on average: 24.7% crude protein, 12.9% crude fat, 4.3% soluble fibre, 18.7% NDF, 0.54% calcium and 0.48% and an energy density of 3.28 kcal/g (13.72 kJ/g), all on a dry matter basis. Water-soluble carbohydrates were determined by calculation and ranged from 60.2 to 34.7% in the typical and improved diets respectively^41^.

### DNA isolation, 16S rRNA gene amplification, and sequencing

We extracted total DNA from each faecal sample using the MoBio PowerSoil DNA isolation kit (MoBio, Carlsbad, CA). We used a two-step PCR protocol to reduce errors and improve overall accuracy^42^. We used KAPA HiFi polymerase for qPCR amplification (Kapa Biosystems, Woburn, MA). We amplified the bacterial 16S rRNA gene using primers 515 F and 806 R, which flanked the V4 hypervariable region of bacterial 16S rRNA genes, following the Earth Microbiome Project protocol. We sequenced amplicons on an Illumina MiSeq sequencer in 2 × 250 paired-end modes^43^.

### Data analysis

We analysed sequence reads using the Quantitative Insights to Microbial Ecology (QIIME) 1.9.1 pipeline^44^. We discarded R2 reads because of low average sequencing quality. We clustered preprocessed sequences *de novo* at 99% nucleotide sequence similarity level. We assigned taxonomies using the BLAST-based “parallel_asign_taxonomy_blast.py” script with GREENGENES_13_8 for assignment of bacterial Operational Taxonomic Units (OTUs) and RIM_DB_14_7 for additional assignments of OTUs matching methanogenic archaea^45–47^. We rarified quality-filtered sequences to 13,500 reads per sample for the downstream analysis. We performed diversity and principal coordinate analyses using QIIME with default settings or using the phyloseq and corrplot R packages^48,49^. Alpha diversity was calculated using Chao1^50^ and Shannon index^51^, and beta diversity was determined using Bray-Curtis dissimilarity^52^. Calculations for linear discriminant analysis effect size (LEFSe) was performed at http://huttenhower.sph.harvard.edu/galaxy/, using p < 0.01 as cut-off and otherwise default settings^53^.

### Functional metagenomic predictions

We used the PICRUSt v1.1.2 pipeline to predict the functional potential of the analysed prokaryotic communities^54^. We clustered sequences used for PICRUSt prediction into OTUs (97% similarity) using the *pick_closed_reference_otus*.*py* QIIME script against the Greengenes database (13_5_release). We discarded any reads that did not hit the reference collection. We used the rarefied OTU table (2,000 sequences per sample) for predicted 16S rRNA gene copy number normalization using the *normalize_by_copy_number*.*py* script, and the functional metagenome profiles prediction using the *predict_metagenomes*.*py* script. We collapsed the resulting table, consisting of Kyoto Encyclopedia of Genes and Genomes (KEGG) Orthologs (KOs) at KO level 3 within the pathway hierarchy of KEGG using the *categorize_by_function*.*py* script. We used the Nearest Sequenced Taxon Index (NSTI) score as an indicator for the accuracy of PICRUSt. We used STAMP^55^, a PCA plot based on Euclidean distances between samples on KEGG level 3 gene ontologies, to visualize the sample clusters and dissimilarity in the predicted composition of functional gene families between diets. We performed analysis of similarity on a Bray-Curtis dissimilarity matrix using the *compare_categories*.*py* QIIME script with 999 permutations.

### Data deposition

We have deposited sequence data to NCBI under PRJNA412965.

## Results

### Diet correlations with shifts in microbiome

**Figure 1:**
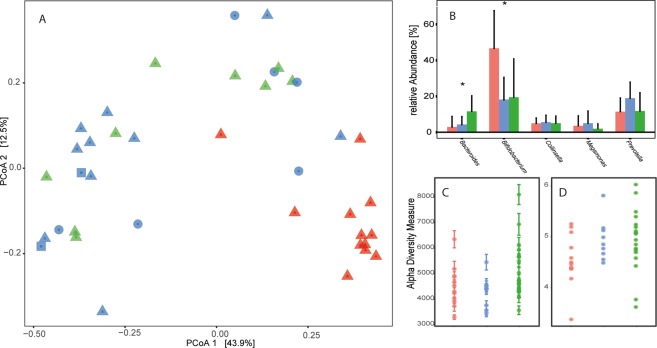
Diet-dependent differences in microbiome composition in *Nycticebus spp*. A PCoA-analysis based on Bray-Curtis dissimilarities of the bacterial microbiota of animals consuming three different diets (red = wild, green = typical diet, blue = improved diet) is shown in panel A. The analysis included animals representing three different *Nycticebus* species (triangles NJ, squares NM, circles NC). The effect of diet on the abundance of the five most abundant genera is shown in panel B. The effect of diet on the abundance of the five most abundant genera is shown in panel B. Statistically significant differences in genera abundance between diets are indicated by asterisk, p < 0.05 (ANOVA with Tukey-Kramer post-hoc test). Panel C and D show the effects of the aforementioned diets on alpha-diversity using two different metrics (Chao1 = panel C, Shannon = panel D).

**Figure 2:**
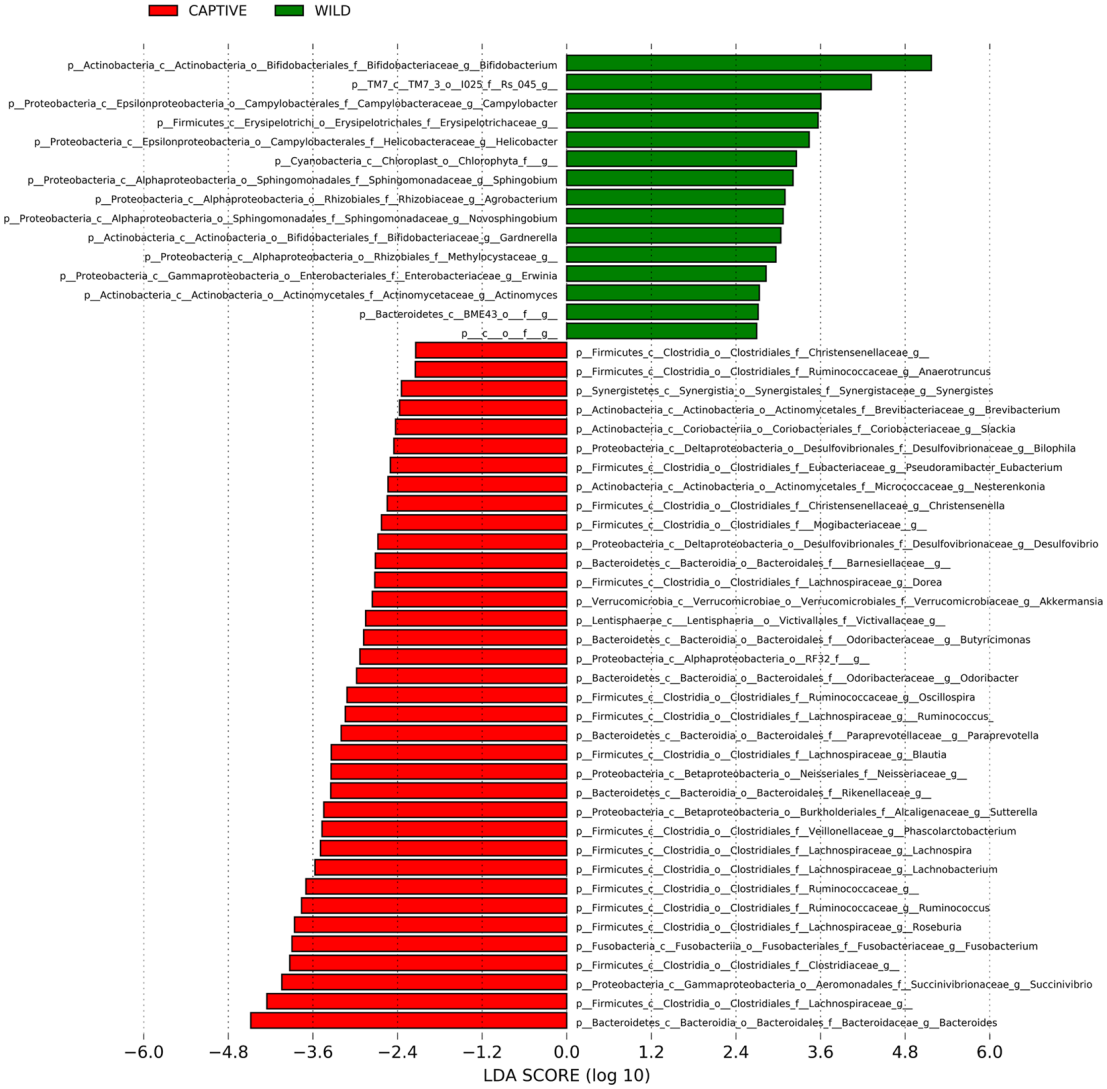
LEFSe-analysis reveals strong microbiome differences between captive and wild slow lorises. Shown are the results from a genus-level analysis that identified the gut microbiome taxa that mostly strongly differed between captive and wild slow lorises (p-value < 0.01, LDA-score >2 or < −2).

Analysis of the gut microbiota revealed strong diet-dependent differences as indicated by principle coordinates analysis based on Bray-Curtis dissimilarities (Fig. 1A). A subsequent analysis of similarity (ANOSIM) of the Bray-Curtis dissimilarity matrix showed that these differences were also statistically significant (p-value = 0.001, R-value = 0.3944, based on 999 permutations). Three different species of *Nycticebus* species were included in this study. No clear separation by *Nycticebus* species was observable under the three different dietary treatments, thus providing evidence that host genetics were not driving the differences in gut microbiota observed between groups (Fig. 1A). A genus-level analysis of the microbiota in the three different groups indicated strong differences between the captive and wild animals (see Table S1 for statistical analysis of differences between genera), but differences between samples from animals on the two different diets were less pronounced (Fig. 1B). *Bifidobacterium* was the most abundant genus in the fecal samples of wild animals and reached average relative abundances of more than 40% (relative abundance ± standard deviation, 46.7% ± 21.0%). We also observed strong differences in mean relative abundance for the genera *Bacteroides* and *Prevotella*, the next most abundant genera in all samples. *Bacteroides* had the highest abundance under the typical diet treatment (11.6% ± 8.7%), while *Prevotella* had the highest abundance on the improved diet (18.9% ± 8.9%). A LEFSe analysis revealed that only one genus (belonging to the Erysipelotrichaceae) out of 268 genera differed statistically significantly between the three different diets (p-value < 0.01 LDA score = 3.96), but that 51 genera differed significantly (p-value < 0.01, LDA score >2 or <−2) between the microbiome of captive and wild animals (Fig. 2).

**Figure 3:**
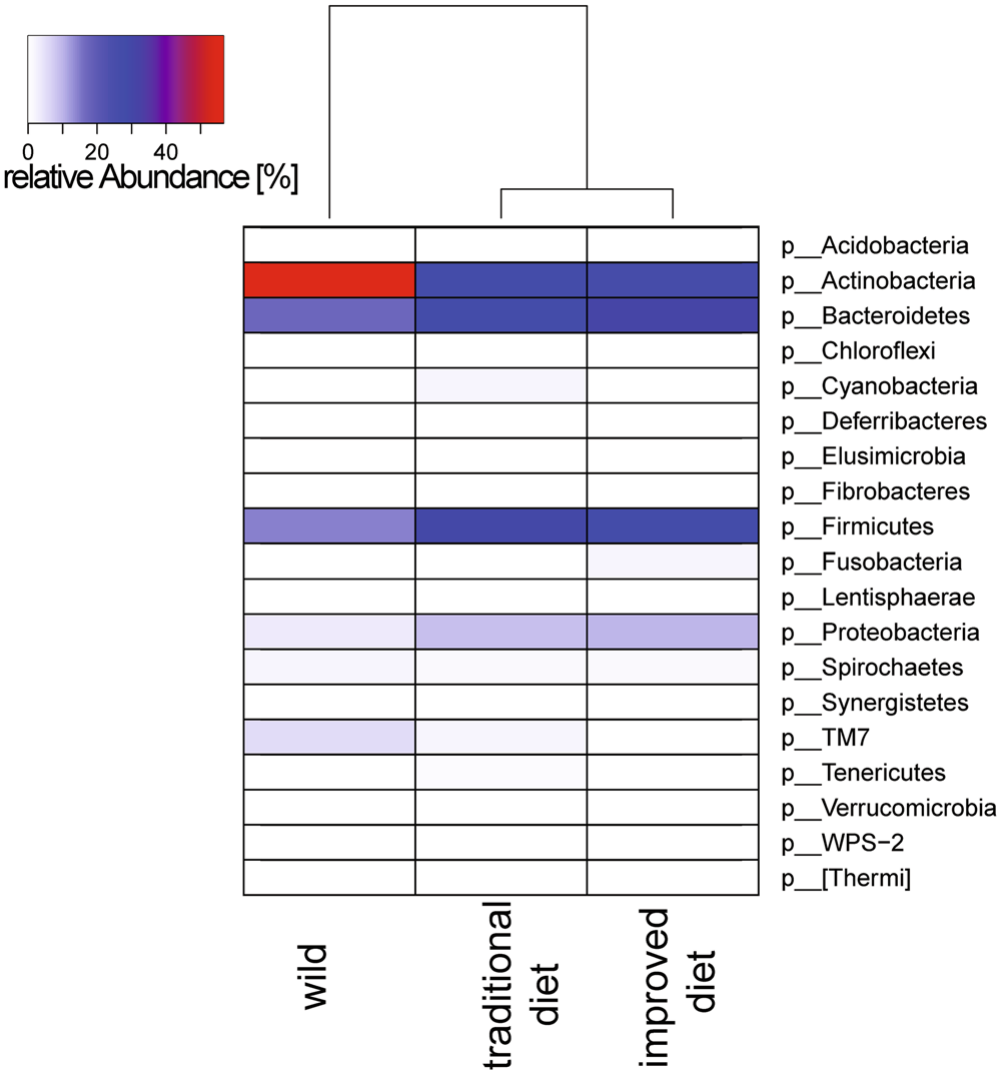
Phylum-level microbiome analysis of slow lorises on different diets. Shown are the data for the 20 most abundant phyla in the slow loris gut microbiome. Averages for each of the groups were calculated prior to clustering.

The differences between samples from the three different diet groups were also apparent at the phylum level (Fig. 3, Table S2). Samples from wild slow lorises showed a higher relative abundance of Actinobacteria (56.7% ± 24.1%), followed by Bacteroidetes (17.1% ± 11.6%), Firmicutes (14.3% ± 9.3%), TM7 (5.2% ± 6.4%), Proteobacteria (4.0% ± 2.6%), Spirochaetes (2.1% ± 4.6%) and other phyla that contributed less than one percent to the relative abundance. The microbiota of the two captive groups were relatively similar to each other. The microbiota of animals on the typical diet was dominated by Bacteroidetes (31.1% ± 16.4%), Firmicutes (27.5% ± 16.7%), Actinobacteria (26.2% ± 25.2%), followed by Proteobacteria (9.8% ± 9.3%), Fusobacteria (2.2% ± 3.9%) and Spirochaetes (1.7% ± 2.0%). Microbiota of animals on the improved diet were dominated by Firmicutes (29.6% ± 8.2%), Bacteroidetes (27.7% ± 8.3%), Actinobacteria (27.0% ± 18.8%), followed by Proteobacteria (8.7% ± 9.1%), TM7 (1.8% ± 2.7%), Cyanobacteria (1.7% ± 3.5%) and Spirochaetes (1.4% ± 1.7%). Other phyla were detected in the faeces of animals on the traditional and improved diets, but these accounted for less than one percent of the relative abundance in the samples. The diets also had an influence on the alpha-diversity as shown in Fig. 1C,D. The analysis of Chao1 and Shannon index show that alpha diversity varies to small degree between the three different treatment groups, but captive animals do not harbour a statistically significant less diverse microbiota than the wild-living animals (based on Kruskal-Wallis rank sum test, p > 0.01).

### Differences in methanogen component of the gut microbiota

**Figure 4:**
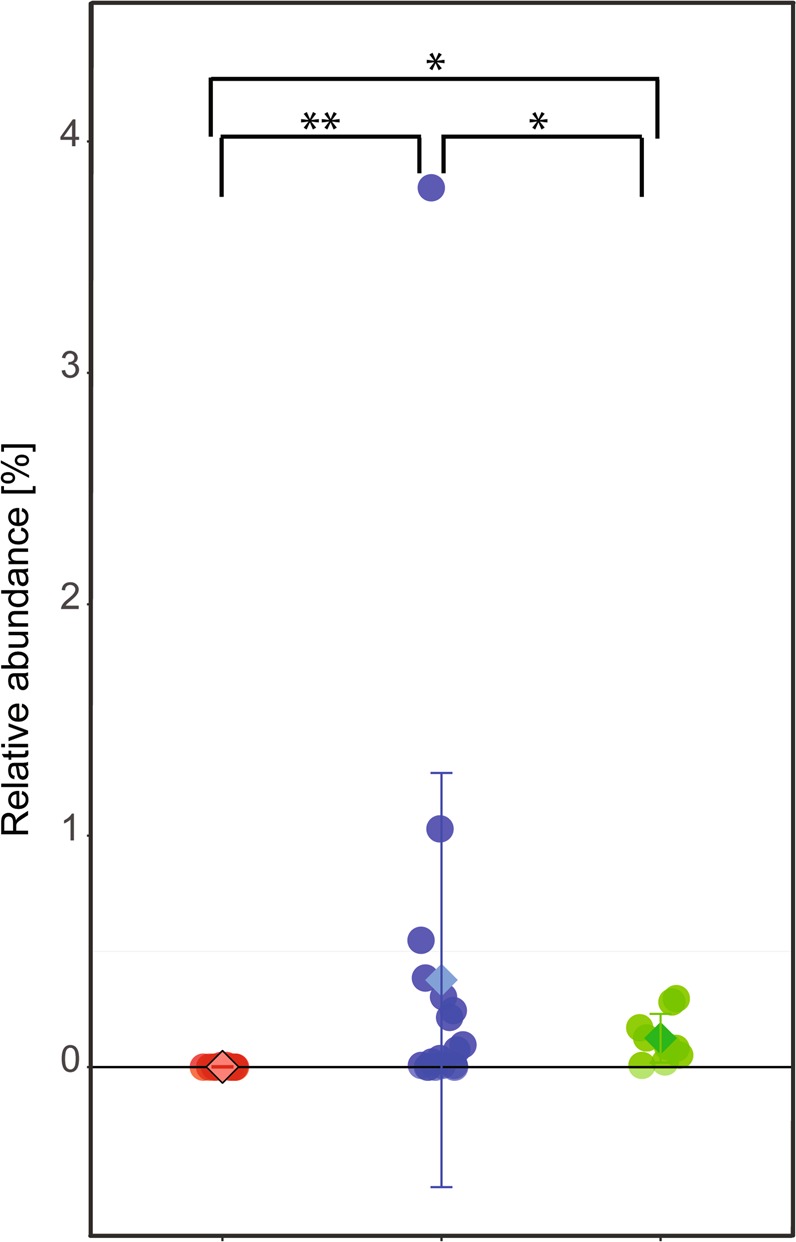
Effect of diet on relative abundance of methanogenic Archaea in *Nycticebus spp*. Shown is the sum of the relative abundances of detected methanogenic archaea per sample in the three different dietary treatment groups (red = wild, green = typical diet, blue = improved diet).

In addition to bacteria, some Archaea were also detectable using 16S rRNA gene sequencing. The majority of detected Archaeal OTUs in this study could be taxonomically assigned as methanogenic Archaea (methanogens), which are a phylogenetically diverse group of microorganisms. Detection of methanogens appeared to be dependent on diet or whether the animals were living in captivity or in the wild. Methanogens were either not detected at all (in nine out 12 samples) or only at low abundance in the remaining three samples (0.007–3.8%) from wild lorises but were detected (with at least one sequence read) in 23 of the 27 samples from captive lorises (Fig. 4). The majority of sequence reads that could be taxonomically assigned were assigned to two orders of methanogens, the Methanobacteriales and Methanomassiliicoccales. The V4 region of 16S rRNA gene of the dominant methanogen OTUs’ sequence identical to that of the human gut isolates, e.g. *Methanobrevibacter smithii* and *Methanosphaera stadtmanae* for Methanobacteriales species, and *Candidatus* Methanomethylophilus alvus for the Methanomassiliicoccales. A low number of reads (<0.1%) were also assignable to OTUs that highest identity to 16S rRNA genes of members of the Methanomicrobia genus *Methanocorpusculum*. It should be noted that the used primer pair may not amplify other archaeal groups that may potentially be present in the intestinal tract of slow lorises.

### Correlations of dietary nutrients and microbial genera

**Figure 5:**
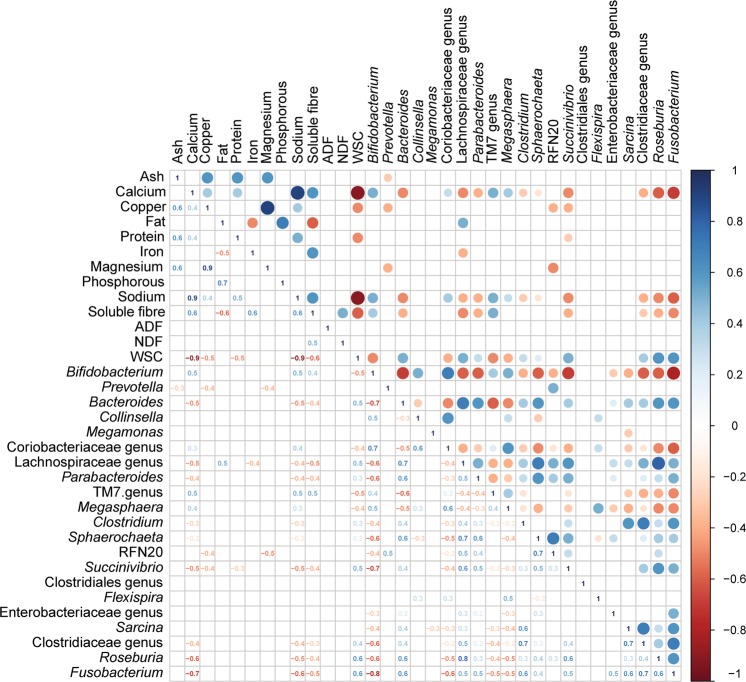
Correlation analysis for dietary nutrients and relative abundance of bacterial genera. Spearman rank correlations were performed followed by Bonferroni corrections. Only statistically significant rho values are shown. Numerical values for each significant correlations are given in the lower triangle.

Using information about the nutrient intake, it was possible to determine how the intake of some nutrients correlates with the abundance of specific microbial taxa (Fig. 5). Of the organic compounds, soluble fiber (SF) and water-soluble carbohydrates (WSC) had the strongest correlations with the 20 most abundant genera. The correlation of SF with relative abundance of the genera was in most cases the inverse of the correlation of genera with WSC (few correlations were not included as they did not meet the threshold after Bonferroni corrections). Other organic dietary contents such as ADF and NDF were not strongly correlated with any of the 20 most abundant genera (Fig. 5).

In addition to correlations between nutrients and taxa, it was also possible to determine correlations between the different genera. The genus *Bifidobacterium* was positively correlated with *Megasphaera* as well as the TM7 genus *Collinsella*, but also strongly negatively correlated (<−0.7) with some genera that have a higher relative abundance in captivity than in wild animals, such as *Bacteroides*, *Fusobacterium*, and *Succinivibrio*.

### Predicted functional metagenomes of the Slow Loris gut microbiome

The NSTI scores were calculated prior to the comparative metagenome analyses to determine the extent to which the detected microorganisms were related to those with already sequenced genomes. NSTI scores across all samples were 6.8% ± 3.7% (expressed as mean ± standard deviation), but no significant differences in NSTI scores between the different dietary groups were observed. The subsequent comparison of predicted functional metagenomes of the slow loris samples indicated that the three different dietary treatments may affect the overall gene content of the gut microbiome (Fig. S1). Analysis of similarity (ANOSIM) of the Bray-Curtis dissimilarity matrix showed that these differences between dietary treatment groups were also statistically significant (p-value = 0.002, R-value = 0.2125, based on 999 permutations). A pairwise comparison between KEGG Level 3 pathways did not reveal statistically significant differences (p-value > 0.05) between samples from animals on typical and improved diet (White’s non-parametric test^56^, Benjamini-Hochberg corrected p-values), but did indicate differences in samples from captive animals from those on the wild diet (see Supporting Table S3 for a full list of pathways). Some of the main differences include a higher abundance of germination and sporulation genes in samples from animals on traditional and improved diets, but also an enrichment of xenobiotic metabolism genes in samples from animals on the wild diet (Fig. S2).

## Discussion

### Javan slow loris microbiome

The gut microbiome of captive slow lorises was significantly different from wild counterparts and feeding them a diet more similar to the wild failed to drive any large-scale changes in the microbial community, which was not expected based on the current literature^57^. The change to the improved diet did nonetheless alter the abundance of some key microbial taxa of wild animals. The wild microbiome of *N*. *javanicus* was largely different from that of the pygmy slow loris (*N*. *pygmaeus*) reported previously^58–60^. Their ecologies are different, with *N*. *pygmaeus* being adapted to seasonal changes in food availability, and a higher intake of insects and nectar than the larger *N*. *javanicus*^61^. We would therefore assume that a larger proportion of the pygmy slow loris diet is composed of WSC and perhaps crude protein. The bacteria necessary to digest the large intake of plant fibres in *N*. *javanicus*, such as Actinobacteria^6^, may not be as useful to *N*. *pygmaeus*. The latter may be more adapted to digesting sugars and complex aromatic compounds, and to a lesser extent, soluble fibres^58^.

The most represented phylum in wild *N*. *javanicus* was Actinobacteria (56%), as opposed to Bacteroidetes for the captive group (26 and 27% for the typical and improved diets, respectively). Actinobacteria has been shown to be in high abundance in other hindgut fermenters such as the gorilla (*Gorilla gorilla gorilla)*. It was described to increase under the influence of a high fat diet, which was not the case for the wild slow lorises^6^. Bacteroidetes are one of the two phyla most well-represented in non-human primate microbiomes^62^, which is consistent with the captive slow lorises examined in this study. The wild slow lorises may be an exception to this rule. This difference could be due to the substantial differences in diet between wild and captive lorises. Further research is necessary to confirm this finding, and to identify the specific contributing dietary compounds. The increase in Bacteroidetes in the captive versus wild individuals is not a surprise, as they tend to increase under the influence of energy-dense diets, such as those higher in fat and sugar^13^.

### Diet change led to a change in microbiota

The diet higher in fibre fractions and lower in WSC led to some changes in the captive slow lorises’ microbiota yet failed to match the microbial fingerprint of wild individuals. Relative abundance of Firmicutes increased under the improved diet but was still much higher than in wild samples. Firmicutes are known to produce a large amount of energy rich short-chain fatty acids^35^. A higher Firmicutes to Bacteroidetes ratio in female black howler monkeys (*Alouatta pigra*) led to higher fermentation efficiencies, and essentially a higher energy assimilation^5^. Firmicutes are also known to include many pectin-degrading species, with pectin being a soluble fibre found in tree gum added to the new captive diet^63^.

Alpha Diversity indices decreased under the improved diet, which was more similar to the wild animals. A large diversity of gut microbes has been described as adaptive and beneficial, driven by a reduction in food diversity and fibre concentration^11^. The wild slow lorises were observed eating between three and five plant food items and a variety of insect species that could not be identified^36,41^. The field site was an agroforest, therefore the plants and animal diversity in the area was lower compared to tropical forests in the vicinity. In comparison, the captive slow lorises on the typical diet had a weekly intake of up to 14 different food items (comprising six to nine fruit species, three insect species, eggs, and occasionally honey), while the improved diet gave them access to a maximum of eight food items (comprising four vegetables, three insect species and an egg). The theory of microbe diversity being tied to food diversity is consistent with our results; however, it was not linked to fibre content. We do not have enough data to surmise if the matching of the new diet and the wild animals in terms of diversity is due solely to food item variety or due to other factors. On a purely analytical level, the new improved diet approached the golden standard but did not reach it.

### Diet change led to a change in microbiome

Wild animals regularly ingested tree gums that were loaded with plant secondary metabolites^36,41^. This dietary complexity is nearly impossible to reproduce in captivity and may also have a significant effect on the microbiome, as many of these microbes contain known detoxification pathways^64^. The functional prediction of the microbiome may support this hypothesis as it indicates an enrichment of genes in the ‘xenobiotic metabolism and degradation’ pathway (Fig. S2). The exact composition of the consumed secondary metabolites and the contributions of the xenobiotic metabolism genes remain to be investigated. The gum fed in the new diet was purified, and thus free of any secondary metabolites.

It should be noted that the calculated NSTI scores were relatively high, indicating that only limited genome information was available for the microorganisms that were detected in the analyses. Additional deep-sequencing efforts of slow loris gut microbiome samples that lead to assembly of high-quality (draft) genomes, as well as cultivation-based approaches to obtain microorganisms from this and similar environments, could help to reduce these limitations. Among the significant differences between the three groups was also an enrichment of germination and sporulation genes in samples from animals on the typical and the improved diets. This difference is congruent with the observation that samples from the same two groups have also a higher representation of Firmicutes than samples from animals on the wild diet. This finding provides additional validation for the functional predictions, despite the relatively low NSTI scores.

### Methanogens as potential biomarkers in wild and captive *Nycticebus* spp.?

The differences in relative abundance of methanogens in the wild and captive slow lorises was unexpected and has certain impact on overall microbiome function. Further studies need to be performed to determine the underlying cause of these differences (e.g. specific dietary metabolites), and also the potential presence of other hydrogen-consuming microorganisms such as acetogens or sulfate-reducers in the gut microbiome of wild slow lorises.

It has been shown in other studies that the abundance of methanogens may increase with host age^65,66^. As an underlying cause for the observations this can be ruled out for the animals analysed in this study. It is striking that the relative abundance of *Bifidobacterium* is significantly higher in the microbiome of wild *Nycticebus spp*. than in the captive counterparts. The higher abundance of Bifidobacteria is often associated with the intestinal microbiota of infants and small children^67,68^ and it could therefore indicate that the natural diet of wild slow lorises may promote a configuration of the microbiome that is more similar to that of young animals/humans, and subsequently leads to lower abundance of methanogens. It may still be premature to say that methanogens can definitely serve as biomarkers; however, this study emphasises that differences in methanogen community structure do exist, and that future studies need to pay more attention to the non-bacterial components of the microbiome.

Lastly, it also needs to be considered that these findings need to be followed-up with more domain-specific primers that target archaea or methanogens specifically. This analysis would allow a more detailed description of the methanogen microbiota in the animals and would also add more sensitivity to the detection of methanogens in the samples. If the low abundance or even absence of methanogens turns out to be a characteristic trait of wild slow lorises, it may also be possible to use this information as a potential biomarker for the effect of diet on the gut microbiota and could be used as rapid PCR-based screening method using specific primers.

## Conclusions

One aim of this study was to use the microbiota analysis as indicator of similarity between captive and wild slow lorises faecal samples. The tested diets in this study do not fully substitute the diet of wild animals, but important lessons for future diet design can be learned, e.g. aiming to increase the relative abundance of Bifidobacteria. The relative abundance of the highly abundant genus *Bifidobacterium* is –among some inorganic ions- positively correlated with soluble fibre. Increasing the content could therefore also help to increase the relative abundance of Bifidobacteria in the gut of captive animals. Increasing soluble fibre and reducing sugar in the diet may lead to shifts in microbe communities which may increase energy harvesting efficiency and have protective effects. Our results were different than what we expected based on the current literature; however, while slow lorises may not be an appropriate model, we did observe some trends which have been universal. The slight shift in microbe community has been associated with both better weight control and better-formed faeces in all animals as well, which may be used as a proxy for improved gut health^41^.

Applying this to all primates and not only slow lorises, it may also be worth considering changing dietary contents to reduce the relative abundance of some microbial taxa. This concern, for example, microbial taxa that have higher abundance in faecal samples of captive animal than in wild animals. Albeit not being statistically significant, the mean relative abundance of the phylum Proteobacteria is more than twice as high in faecal samples from captive animals as in wild animals. This phylum contains several pathogenic and opportunistic pathogenic microorganisms. Among the genera detected in the slow lorises faeces are members of the *Helicobacter (*assigned as *‘Flexispira’* by Greengenes, but this genus name is not validly published and OTUs taxonomically assigned as *Flexispira* should be considered to belong to the genus *Helicobacter*^69^), *Bilophila* and less well-characterised Enterobactericeae genera. Whether this high relative abundance of the Proteobacteria is an indicator of increased susceptibility to infections or simply a strong deviation from the composition of native microbiome is speculative and will require additional research. However, correlation analysis suggests that reducing the contents of WSC could help to reduce the relative abundance of some Proteobacteria and concomitantly the risk of infections. However, not all Proteobacteria taxa seem to follow this same pattern and follow-up studies with additional and/or more defined diets will be required to further improve the composition of slow lorises diets. Lastly, it needs to be noted that understanding the underlying mechanisms of dietary interventions will require additional attention. The observed correlations could simply indicate a co-occurrence of genera and/or substrates, but it may also point towards a diet-specific metabolic network with syntrophic interaction between its members.

## Supplementary information

Figure S1-2 Table S1-3

## References

[1] Doran‐Sheehy D, Mongo P, Lodwick J, Conklin‐Brittain N (2009). Male and female western gorilla diet: preferred foods, use of fallback resources, and implications for ape versus old world monkey foraging strategies. American Journal of Physical Anthropology.

[2] Crimmins SM, Roberts NM, Hamilton DA (2009). Effects of prey size on scat analysis to determine river otter *Lontra canadensis* diet. Wildlife Biology.

[3] Knott CD (1998). Changes in orangutan caloric intake, energy balance, and ketones in response to fluctuating fruit availability. International Journal of Primatology.

[4] Srivathsan A, Sha J, Vogler AP, Meier R (2015). Comparing the effectiveness of metagenomics and metabarcoding for diet analysis of a leaf‐feeding monkey (*Pygathrix nemaeus*). Molecular Ecology Resources.

[5] Amato KR (2014). The role of gut microbes in satisfying the nutritional demands of adult and juvenile wild, black howler monkeys (*Alouatta pigra*). American Journal of Physical Anthropology.

[6] Gomez A (2016). Temporal variation selects for diet–microbe co-metabolic traits in the gut of Gorilla spp. The ISME journal.

[7] McKenzie VJ (2017). The Effects of Captivity on the Mammalian Gut Microbiome. Integrative and Comparative Biology.

[8] Cho I, Blaser MJ (2012). The human microbiome: at the interface of health and disease. Nature Reviews Genetics.

[9] Clemente JC, Ursell LK, Parfrey LW, Knight R (2012). The impact of the gut microbiota on human health: an integrative view. Cell.

[10] Goodrich JK (2014). Human genetics shape the gut microbiome. Cell.

[11] Clayton JB (2016). Captivity humanizes the primate microbiome. Proceedings of the National Academy of Sciences.

[12] David LA (2014). Diet rapidly and reproducibly alters the human gut microbiome. Nature.

[13] Wu GD (2011). Linking long-term dietary patterns with gut microbial enterotypes. Science.

[14] Trayford HR, Farmer KH (2013). Putting the spotlight on internally displaced animals (IDAs): a survey of primate sanctuaries in Africa, Asia, and the Americas. American Journal of Primatology.

[15] Hooper LV, Littman DR, Macpherson AJ (2012). Interactions between the microbiota and the immune system. Science.

[16] Buffie CG (2015). Precision microbiome reconstitution restores bile acid mediated resistance to *Clostridium difficile*. Nature.

[17] Buffie CG, Pamer EG (2013). Microbiota-mediated colonization resistance against intestinal pathogens. Nature Reviews Immunology.

[18] Hacquard S (2015). Microbiota and host nutrition across plant and animal kingdoms. Cell Host & Microbe.

[19] Turnbaugh PJ (2006). An obesity-associated gut microbiome with increased capacity for energy harvest. Nature.

[20] Petersen C, Round JL (2014). Defining dysbiosis and its influence on host immunity and disease. Cellular Microbiology.

[21] Redford KH, Jensen DB, Breheny JJ (2012). Integrating the captive and the wild. Science.

[22] Amato KR (2015). The gut microbiota appears to compensate for seasonal diet variation in the wild black howler monkey (*Alouatta pigra*). Microbial Ecology.

[23] Nijman, V., Shepherd, C. R. & Nekaris, K. A.-I. Trade in Bengal slow lorises in Mong La, Myanmar, on the China border. *Primate Conservation*, 139–142 (2014).

[24] Ratajszczak RT (1998). distribution and status of the lesser slow loris *Nycticebus pygmaeus* and their implications for captive management. Folia Primatologica.

[25] Shepherd, C. R., Sukumaran, J. & Wich, S. A. *Open season: An analysis of the pet trade in Medan*, *Sumatra*, *1997–2001*. (TRAFFIC Southeast Asia, 2004).

[26] Cabana F, Nekaris K (2015). Diets high in fruits and low in gum exudates promote the occurrence and development of dental disease in pygmy slow loris (*Nycticebus pygmaeus*). Zoo Biology.

[27] Collins, R. & Nekaris, K. Release of greater slow lorises, confiscated from the pet trade, to Batutegi Protected Forest, Sumatra, Indonesia. *Global re-introduction perspectives*. *IUCN Reintroduction Specialist Group*, *Abu Dhabi*, 192–195 (2008).

[28] Kenyon M (2014). Survival of reintroduced pygmy slow loris *Nycticebus pygmaeus* in South Vietnam. *Endangered Species*. Research.

[29] Moore R, Nekaris K (2014). Compassionate conservation, rehabilitation and translocation of Indonesian slow lorises. *Endangered Species*. Research.

[30] Poindexter S, Khoa D, Nekaris K (2017). Ranging patterns of reintroduced pygmy slow lorises (Nycticebus pygmaeus) in Cuc Phuong National Park, Vietnam. Vietnamese Journal of Primatology.

[31] Streicher U, Nadler T (2003). Re-introduction of pygmy lorises in Vietnam. Reintroduction News.

[32] van der Sandt, L. Towards a successful translocation of captive slow lorises (*Nycticebus* spp.) in Borneo: a review and recommendations. *bioRxiv*, 078535 (2016).

[33] Soorae, P. S. *Global re-introduction perspectives: re-introduction case-studies from around the globe*. (Iucn, 2008).

[34] Fogel AT (2015). The gut microbiome of wild lemurs: a comparison of sympatric *Lemur catta* and *Propithecus verreauxi*. Folia Primatologica.

[35] Schleifer, K.-H. In *Bergey’s Manual® of Systematic Bacteriology* 19–1317 (Springer, 2009).

[36] Cabana F, Dierenfeld E, Wirdateti W, Donati G, Nekaris K (2017). The seasonal feeding ecology of the Javan slow loris (Nycticebus javanicus). American Journal of Physical Anthropology.

[37] Nekaris K (2014). Extreme primates: Ecology and evolution of Asian lorises. Evolutionary Anthropology: Issues, News, and Reviews.

[38] Cabana F, Dierenfeld ES, Donati G, Nekaris K (2018). Exploiting a readily available but hard to digest resource: a review of exudativorous mammals identified thus far and how they cope in captivity. Integrative zoology.

[39] Albenberg LG, Wu GD (2014). Diet and the intestinal microbiome: associations, functions, and implications for health and disease. Gastroenterology.

[40] Rode-Margono EJ, Nijman V, Wirdateti NK, Nekaris K (2014). Ethology of the critically endangered Javan slow loris *Nycticebus javanicus* E. Geoffroy Saint-Hilaire in West Java. *Asian*. Primates.

[41] Cabana, F., Dierenfeld, E., Wirdateti, W., Donati, G. & Nekaris, K. Trialling nutrient recommendations for slow lorises (*Nycticebus* spp.) based on wild feeding ecology. *Journal of Animal Physiology and Animal Nutrition* (2017). 10.1111/jpn.12694 28444791

[42] Gohl DM (2016). Systematic improvement of amplicon marker gene methods for increased accuracy in microbiome studies. Nature Biotechnology.

[43] Thompson, L. R. *et al*. A communal catalogue reveals Earth’s multiscale microbial diversity. *Nature* **551** (2017). 10.1038/nature24621 PMC6192678 29088705

[44] Caporaso JG (2010). QIIME allows analysis of high-throughput community sequencing data. Nature Methods.

[45] Altschul SF (1997). Gapped BLAST and PSI-BLAST: a new generation of protein database search programs. Nucleic Acids Research.

[46] McDonald D (2012). An improved Greengenes taxonomy with explicit ranks for ecological and evolutionary analyses of bacteria and archaea. The ISME Journal.

[47] Seedorf H, Kittelmann S, Henderson G, Janssen PH (2014). RIM-DB: a taxonomic framework for community structure analysis of methanogenic archaea from the rumen and other intestinal environments. PeerJ.

[48] McMurdie PJ, Holmes S (2013). phyloseq: an R package for reproducible interactive analysis and graphics of microbiome census data. PloS one.

[49] Wei T (2017). Package ‘corrplot’. Statistician.

[50] Chao, A. Nonparametric estimation of the number of classes in a population. *Scandinavian Journal of statistics*, 265–270 (1984).

[51] Shannon CE (2001). A mathematical theory of communication. ACM SIGMOBILE Mobile Computing and Communications Review.

[52] Bray JR, Curtis JT (1957). An ordination of the upland forest communities of southern Wisconsin. Ecological monographs.

[53] Segata N (2011). Metagenomic biomarker discovery and explanation. Genome biology.

[54] Langille MG (2013). Predictive functional profiling of microbial communities using 16S rRNA marker gene sequences. Nature Biotechnology.

[55] Parks DH, Tyson GW, Hugenholtz P, Beiko RG (2014). STAMP: statistical analysis of taxonomic and functional profiles. Bioinformatics.

[56] White JR, Nagarajan N, Pop M (2009). Statistical methods for detecting differentially abundant features in clinical metagenomic samples. PLoS Computational Biology.

[57] Clayton, J. B. *et al*. The gut microbiome of nonhuman primates: Lessons in ecology and evolution. *American journal of primatology*, e22867 (2018). 10.1002/ajp.22867 29862519

[58] Bo X (2010). Phylogenetic analysis of the fecal flora of the wild pygmy loris. American Journal of Primatology.

[59] Xu B (2013). Metagenomic analysis of the pygmy loris fecal microbiome reveals unique functional capacity related to metabolism of aromatic compounds. PLoS One.

[60] Xu B (2014). Cloning and characterization of a novel alpha-amylase from a fecal microbial metagenome. Journal of Microbiology and Biotechnology.

[61] Starr C, Nekaris K (2013). Obligate exudativory characterizes the diet of the pygmy slow loris *Nycticebus pygmaeus*. American Journal of Primatology.

[62] Yildirim S (2010). Characterization of the fecal microbiome from non-human wild primates reveals species specific microbial communities. PloS one.

[63] Cornick NA, Jensen N, Stahl D, Hartman P, Allison M (1994). *Lachnospira pectinoschiza* sp. nov., an anaerobic pectinophile from the pig intestine. International Journal of Systematic and Evolutionary Microbiology.

[64] Nicholson JK (2012). Host-gut microbiota metabolic interactions. Science.

[65] Maczulak AE, Wolin M, Miller TL (1989). Increase in colonic methanogens and total anaerobes in aging rats. Applied and Environmental Microbiology.

[66] Vanderhaeghen S, Lacroix C, Schwab C (2015). Methanogen communities in stools of humans of different age and health status and co-occurrence with bacteria. FEMS Microbiology Letters.

[67] Azad MB (2013). Gut microbiota of healthy Canadian infants: Profiles by mode of delivery and infant diet at 4 months. Canadian Medical Association Journal.

[68] Yatsunenko T (2012). Human Gut Microbiome Viewed Across Age and Geography. Nature.

[69] Hänninen M-L, Kärenlampi R, Koort J, Mikkonen T, Björkroth K (2005). Extension of the species Helicobacter bilis to include the reference strains of Helicobacter sp. flexispira taxa 2, 3 and 8 and Finnish canine and feline flexispira strains. International journal of systematic and evolutionary microbiology.

