# Supplementary material for: Nutrient-based diet modifications impact on the gut microbiome of the Javan slow loris (Nycticebus javanicus)
Source: Sci Rep. 2019 Mar 11;9:4078. doi: 10.1038/s41598-019-40911-0 (PMC6411731; doi:10.1038/s41598-019-40911-0)
Supplement: Supplementary file 1 — Figure S1-2 Table S1-3 [file 41598_2019_40911_MOESM1_ESM.pdf]

Supplemental information

Nutrient-based diet modifications impact on the gut microbiome of the Javan slow loris (*Nycticebus javanicus*)

Authors: F Cabana[1,2,3], J Clayton[3,4,5,6], KAI Nekaris[2], W Wirdateti[7], D Knights[3,4,6] , H Seedorf[8,9]

[1]Wildlife Nutrition Centre, Wildlife Reserves Singapore, 80 Mandai Lake Road, 729826, Singapore

[2]Nocturnal Primate Research Group, Oxford Brookes University, Gypsy Lane, Oxford, OX3 0BP

[3]Primate Microbiome Project, 6-124 MCB, 420 Washington Ave SE, Minneapolis, MN 55455, USA

[4]Department of Computer Science and Engineering, University of Minnesota, 4-192 Keller Hall, 200 Union St SE, Minneapolis, MN 55455, USA

[5]GreenViet Biodiversity Conservation Center, K39/21 Thanh Vinh Street, Son Tra District, Danang, Vietnam

[6]Biotechnology Institute, University of Minnesota, 1479 Gortner Avenue, Saint Paul, MN 55108, USA

[7]LIPI Zoology, Jalan Raya Bogor KM.46, Kel. Nanggewer Mekar, Kec. Cibinong, Cibinong, Bogor, Jawa Barat 16911, Indonesia

[8]Temasek Life Sciences Laboratory, 1 Research Link, Singapore 117604, Singapore

[9]Department of Biological Sciences, National University of Singapore, Singapore 117604, Singapore

Contact information: Francis Cabana PhD, 80 Mandai Lake Road, 729826, Singapore. E-mail: Francis.cabana@wrs.com.sg

Henning Seedorf, 1 Research Link, Singapore 117604, Singapore, email: henning@tll.org.sg

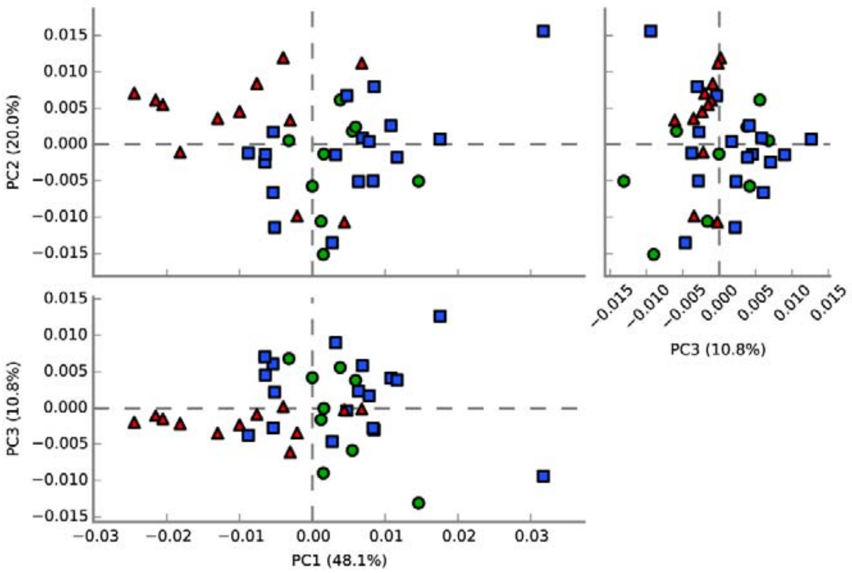

Figure S1. Diet-dependent differences in gene content in the gut microbiome of *Nycticebus spp.* PCA plot based on Euclidean distances between samples of animals consuming three different diets (red=wild, captive: green=improved diet and blue=traditional diet).

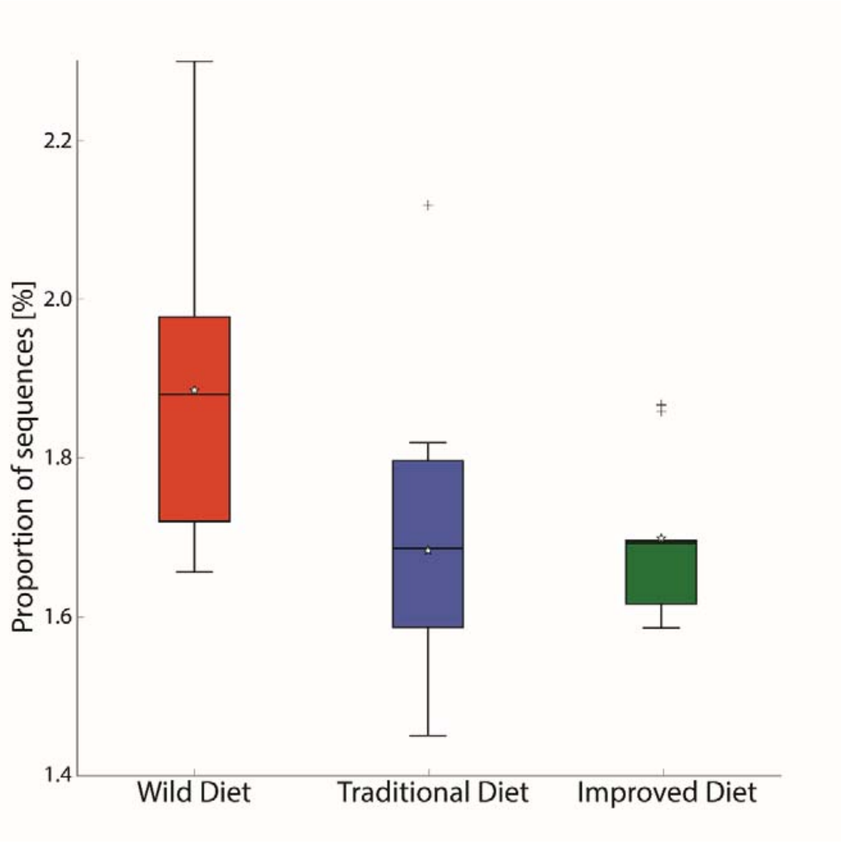

Figure S2. The microbiome of slow lorises on the wild diet is predicted to be enriched for xenobiotics metabolism. Shown are the results for the Level 2 KEGG pathway "Xenobiotics Biodegradation and Metabolism". Statistical significance was determined by performing a Kruskal-Wallis test (Benjamini-Hochberg adjusted p-value=0.016).

Table S1. Genus-level analysis of slow loris microbiota. Correcting p-values have undergone Benjamini-Hochberg Correction.

Level_1	Level_6	p-values	p-values (corrected)	Effect size	Traditional Diet: mean rel. freq. (%) ± std. dev. (%)	Wild Diet: mean rel. freq. (%) ± std. dev. (%)	Improved Diet: mean rel. freq. (%) ± std. dev. (%)
k__Bacteria	g__[Eubacterium]	8.047E-01	8.367E-01	1.200E-02	0.1426±0.2263	0.0972±0.1029	0.1277±0.1419
k__Bacteria	g__[Prevotella]	3.595E-01	6.448E-01	5.525E-02	0.5366±1.3018	0.0529±0.0319	0.2158±0.154
k__Bacteria	g__[Ruminococcus]	9.365E-03	2.688E-01	2.286E-01	0.4708±0.4933	0.0135±0.0118	0.2122±0.3044
k__Bacteria	g__02d06	3.416E-01	6.580E-01	5.793E-02	0.0014±0.0043	0±0	0±0
k__Bacteria	g__1-68	3.434E-01	6.571E-01	5.765E-02	0.0028±0.0084	0±0	0±0
k__Bacteria	g__5-7N15	5.705E-01	7.213E-01	3.070E-02	0.0013±0.0055	0±0	0±0
k__Bacteria	g__A17	1.916E-01	6.545E-01	8.772E-02	0±0	0±0	0.0013±0.0037
k__Bacteria	g__Acetobacter	4.485E-02	4.767E-01	1.584E-01	0.0005±0.0021	0±0	0.0087±0.0167
k__Bacteria	g__Achromobacter	3.525E-01	6.486E-01	5.628E-02	0.0016±0.0049	0±0	0±0
k__Bacteria	g__Acidovorax	3.333E-01	7.139E-01	5.921E-02	0±0	0.0007±0.0023	0±0
k__Bacteria	g__Acinetobacter	7.052E-01	7.580E-01	1.922E-02	0.4083±1.572	0.3592±0.8002	0.0077±0.0093
k__Bacteria	g__Actinobacillus	9.701E-02	6.960E-01	1.216E-01	0±0	0.003±0.0068	0±0
k__Bacteria	g__Actinomyces	1.955E-02	3.507E-01	1.964E-01	0.0037±0.0057	0.1283±0.2083	0.0059±0.0091
k__Bacteria	g__Actinomycetospora	3.543E-01	6.435E-01	5.602E-02	0.0009±0.0037	0.0033±0.0085	0±0
k__Bacteria	g__Adlercreutzia	7.361E-02	6.402E-01	1.349E-01	0.1241±0.1217	0.3001±0.237	0.218±0.2389
k__Bacteria	g__Aerococcus	2.745E-01	7.432E-01	6.931E-02	0.0036±0.0112	0.0393±0.1022	0.0079±0.0224
k__Bacteria	g__AF12	7.485E-01	7.869E-01	1.597E-02	0.001±0.0041	0.0007±0.0023	0±0
k__Bacteria	g__Aggregatibacter	5.009E-01	7.526E-01	3.768E-02	0.008±0.0193	0.0016±0.0036	0.0061±0.0091
k__Bacteria	g__Agrobacterium	6.500E-02	5.829E-01	1.409E-01	0±0	0.0145±0.0298	0±0
k__Bacteria	g__Agrococcus	3.517E-01	6.513E-01	5.640E-02	0.0005±0.0022	0.0016±0.0036	0±0
k__Bacteria	g__Agromyces	5.705E-01	7.244E-01	3.070E-02	0.0004±0.0018	0±0	0±0
k__Bacteria	g__Akkermansia	4.010E-01	6.810E-01	4.950E-02	0.1534±0.3774	0.0079±0.0133	0.0956±0.1993
k__Bacteria	g__Alcanivorax	5.705E-01	8.186E-01	3.070E-02	0.0005±0.002	0±0	0±0
k__Bacteria	g__Alicyclobacillus	5.705E-01	7.723E-01	3.070E-02	0.0009±0.0037	0±0	0±0
k__Bacteria	g__Alkalibacterium	5.705E-01	8.065E-01	3.070E-02	0.0004±0.0018	0±0	0±0
k__Bacteria	g__Allobaculum	9.619E-01	9.686E-01	2.157E-03	0.0017±0.0072	0.0014±0.003	0.0011±0.0032
k__Bacteria	g__Alloiococcus	5.705E-01	7.442E-01	3.070E-02	0.0022±0.0092	0±0	0±0
k__Bacteria	g__Amaricoccus	1.916E-01	6.393E-01	8.772E-02	0±0	0±0	0.0023±0.0064
k__Bacteria	g__Anaerococcus	4.324E-01	7.012E-01	4.551E-02	0.0058±0.0179	0±0	0.0011±0.0032
k__Bacteria	g__Anaerofilum	3.333E-01	6.508E-01	5.921E-02	0±0	0.0008±0.0026	0±0
k__Bacteria	g__Anaerofustis	1.916E-01	7.854E-01	8.772E-02	0±0	0±0	0.0024±0.0067
k__Bacteria	g__Anaeromyxobacter	5.705E-01	7.796E-01	3.070E-02	0.0009±0.0036	0±0	0±0
k__Bacteria	g__Anaerospora	3.333E-01	7.303E-01	5.921E-02	0±0	0.0008±0.0026	0±0
k__Bacteria	g__Anaerostipes	5.985E-01	6.926E-01	2.811E-02	0.0005±0.0019	0.0015±0.0034	0.0011±0.0032
k__Bacteria	g__Anaerotruncus	1.512E-01	7.888E-01	9.964E-02	0.0411±0.0863	0±0	0.0048±0.0091
k__Bacteria	g__Anaerovibrio	7.042E-01	7.598E-01	1.930E-02	0.0004±0.0018	0.0007±0.0023	0±0
k__Bacteria	g__Anaerovorax	2.576E-01	7.469E-01	7.258E-02	0.002±0.0038	0±0	0.0013±0.0037
k__Bacteria	g__Anoxybacillus	5.332E-01	7.807E-01	3.434E-02	0.0005±0.0019	0±0	0.0009±0.0027
k__Bacteria	g__Arthrobacter	5.887E-01	6.869E-01	2.900E-02	0.0552±0.199	0.0075±0.0144	0.0092±0.0131
k__Bacteria	g__Bacillus	1.953E-01	6.442E-01	8.675E-02	0.0125±0.0208	0.003±0.0043	0.0341±0.0714
k__Bacteria	g__Bacteroides	9.760E-03	2.547E-01	2.268E-01	13.4369±9.6263	3.6769±6.8266	6.214±6.4227
k__Bacteria	g__Bdellovibrio	5.705E-01	7.476E-01	3.070E-02	0.0004±0.0018	0±0	0±0
k__Bacteria	g__Bifidobacterium	3.244E-04	2.327E-02	3.600E-01	21.3762±21.854	54.7719±22.2101	22.5757±13.2393
k__Bacteria	g__Bilophila	4.345E-03	2.494E-01	2.608E-01	0.0159±0.0177	0±0	0.0035±0.005
k__Bacteria	g__Blautia	8.849E-03	2.822E-01	2.310E-01	0.4674±0.6119	0.022±0.0163	0.777±0.6231
k__Bacteria	g__Bombiscardovia	3.333E-01	7.533E-01	5.921E-02	0±0	0.0008±0.0027	0±0
k__Bacteria	g__Brachybacterium	2.727E-01	7.526E-01	6.964E-02	0.0191±0.0421	0.0031±0.0057	0.0034±0.0048
k__Bacteria	g__Brachyspira	5.705E-01	8.146E-01	3.070E-02	0.0004±0.0018	0±0	0±0
k__Bacteria	g__Brevibacterium	1.328E-01	7.192E-01	1.061E-01	0.0459±0.088	0.0014±0.0032	0.0092±0.0119
k__Bacteria	g__Brevundimonas	3.096E-01	7.863E-01	6.306E-02	0.0011±0.0031	0±0	0±0
k__Bacteria	g__Bulleidia	2.333E-01	6.902E-01	7.768E-02	0.0019±0.0035	0±0	0.0011±0.003
k__Bacteria	g__Burkholderia	3.333E-01	6.882E-01	5.921E-02	0±0	0.0016±0.0054	0±0
k__Bacteria	g__Butyricimonas	1.783E-02	3.412E-01	2.005E-01	0.3082±0.4032	0.0007±0.0023	0.0765±0.1133
k__Bacteria	g__Butyrivibrio	2.509E-02	3.790E-01	1.851E-01	0.4187±0.4149	0.1081±0.0728	0.2175±0.0919
k__Bacteria	g__Caloramator	1.916E-01	6.872E-01	8.772E-02	0±0	0±0	0.0028±0.008
k__Bacteria	g__Campylobacter	7.363E-02	6.215E-01	1.349E-01	0.4837±1.0593	1.3774±1.6741	0.2193±0.1536
k__Bacteria	g__Candidatus Arthromitus	5.705E-01	7.181E-01	3.070E-02	0.0029±0.0119	0±0	0±0
k__Bacteria	g__Candidatus Koribacter	5.705E-01	7.615E-01	3.070E-02	0.0004±0.0018	0±0	0±0
k__Bacteria	g__Candidatus Portiera	4.076E-01	6.801E-01	4.864E-02	0±0	0.0007±0.0023	0.0009±0.0026
k__Bacteria	g__Capnocytophaga	9.636E-02	7.091E-01	1.219E-01	0.0005±0.0022	0±0	0.0035±0.0069
k__Bacteria	g__Cardiobacterium	2.851E-02	3.896E-01	1.793E-01	0±0	0±0	0.0024±0.0046
k__Bacteria	g__Catenibacterium	2.801E-01	7.513E-01	6.826E-02	0.0587±0.1119	0.0069±0.0065	0.0875±0.1732
k__Bacteria	g__Catonella	5.705E-01	7.342E-01	3.070E-02	0.0005±0.0021	0±0	0±0
k__Bacteria	g__Cellulosimicrobium	6.851E-01	7.476E-01	2.079E-02	0.0016±0.0067	0±0	0.0013±0.0037
k__Bacteria	g__Cetobacterium	7.070E-01	7.543E-01	1.908E-02	0.0005±0.0019	0.0007±0.0023	0±0
k__Bacteria	g__CF231	6.880E-01	7.480E-01	2.056E-02	0.0009±0.0038	0.0023±0.004	0.0019±0.0053
k__Bacteria	g__Christensenella	1.076E-01	6.864E-01	1.165E-01	0.0173±0.0315	0±0	0.0041±0.0078
k__Bacteria	g__Chryseobacterium	3.910E-01	6.760E-01	5.083E-02	0.0084±0.0268	0±0	0±0
k__Bacteria	g__Citricoccus	2.023E-01	6.525E-01	8.494E-02	0.0027±0.0065	0±0	0±0
k__Bacteria	g__Citrobacter	1.345E-01	7.151E-01	1.055E-01	0.0005±0.0019	0.0015±0.0034	0.0166±0.04
k__Bacteria	g__Cloacibacterium	3.333E-01	7.034E-01	5.921E-02	0±0	0.0021±0.0069	0±0
k__Bacteria	g__Clostridium	5.981E-01	6.950E-01	2.815E-02	3.6021±8.5382	1.0116±2.0934	2.2915±5.9736
k__Bacteria	g__Cohnella	5.705E-01	7.309E-01	3.070E-02	0.0004±0.0018	0±0	0±0
k__Bacteria	g__Collinsella	7.768E-01	8.107E-01	1.393E-02	6.1175±4.54	6.1646±3.6738	7.3426±4.4844
k__Bacteria	g__Comamonas	2.945E-01	7.754E-01	6.566E-02	0.0037±0.0112	0.0255±0.0716	0±0
k__Bacteria	g__Coprobacillus	5.193E-01	7.763E-01	3.574E-02	0.1006±0.369	0±0	0.009±0.0256
k__Bacteria	g__Coprococcus	6.688E-01	7.382E-01	2.210E-02	0.3592±0.7687	0.5347±1.2435	0.1721±0.3143
k__Bacteria	g__Coriobacterium	3.539E-01	6.470E-01	5.607E-02	0.001±0.0027	0.0015±0.0035	0.0037±0.0072
k__Bacteria	g__Corynebacterium	6.511E-01	7.329E-01	2.355E-02	0.0782±0.1656	0.0531±0.0909	0.03±0.0397
k__Bacteria	g__Curtobacterium	6.579E-01	7.347E-01	2.299E-02	0.0004±0.0018	0.0008±0.0028	0±0
k__Bacteria	g__Cytophaga	3.333E-01	6.983E-01	5.921E-02	0±0	0.0016±0.0053	0±0
k__Bacteria	g__DA101	5.705E-01	7.088E-01	3.070E-02	0.0004±0.0018	0±0	0±0
k__Bacteria	g__Dehalobacterium	2.173E-01	6.778E-01	8.132E-02	0.0084±0.016	0.0015±0.0033	0.0019±0.0053
k__Bacteria	g__Deinococcus	5.705E-01	7.057E-01	3.070E-02	0.0005±0.0021	0±0	0±0
k__Bacteria	g__Delftia	6.279E-01	7.180E-01	2.552E-02	0.001±0.0028	0.0008±0.0028	0±0
k__Bacteria	g__Dermacoccus	5.705E-01	7.948E-01	3.070E-02	0.0005±0.0021	0±0	0±0
k__Bacteria	g__Desulfosporosinus	1.916E-01	6.959E-01	8.772E-02	0±0	0±0	0.0011±0.003
k__Bacteria	g__Desulfovibrio	2.563E-02	3.677E-01	1.842E-01	0.1524±0.2093	0.0015±0.0034	0.0427±0.0368
k__Bacteria	g__Devosia	1.868E-01	8.649E-01	8.898E-02	0.0009±0.0027	0.0053±0.0122	0±0
k__Bacteria	g__Dialister	7.241E-01	7.668E-01	1.778E-02	0.8019±1.1388	1.0407±0.7592	1.1041±0.9865
k__Bacteria	g__Dietzia	4.404E-01	7.021E-01	4.454E-02	0.0031±0.0097	0±0	0.0045±0.0097
k__Bacteria	g__Dokdonella	3.333E-01	6.643E-01	5.921E-02	0±0	0.0008±0.0027	0±0
k__Bacteria	g__Dorea	7.447E-03	3.053E-01	2.383E-01	0.1759±0.1882	0.0084±0.0071	0.0594±0.0824
k__Bacteria	g__Dysgonomonas	3.482E-01	6.574E-01	5.693E-02	0.0035±0.0089	0±0	0.0013±0.0037
k__Bacteria	g__Eggerthella	3.444E-01	6.545E-01	5.750E-02	0.0053±0.0129	0.0008±0.0025	0.0009±0.0026
k__Bacteria	g__Eikenella	1.916E-01	8.330E-01	8.772E-02	0±0	0±0	0.0011±0.0032
k__Bacteria	g__Enterobacter	9.229E-01	9.392E-01	4.449E-03	0.0019±0.006	0.0014±0.0031	0.0022±0.0043
k__Bacteria	g__Enterococcus	3.855E-01	6.706E-01	5.157E-02	0.1179±0.1716	0.3493±0.7454	0.1582±0.1639
k__Bacteria	g__Epulopiscium	6.142E-01	7.050E-01	2.672E-02	1.3125±5.2109	0.2425±0.5873	0.0106±0.0171
k__Bacteria	g__Erwinia	2.081E-02	3.513E-01	1.936E-01	0.0015±0.0047	0.0244±0.036	0.0039±0.011
k__Bacteria	g__Erysipelothrix	6.752E-01	7.425E-01	2.158E-02	0.0005±0.0019	0.0008±0.0026	0±0
k__Bacteria	g__Euzebya	3.333E-01	6.690E-01	5.921E-02	0±0	0.0008±0.0027	0±0
k__Bacteria	g__Facklamia	2.725E-01	7.593E-01	6.968E-02	0.0063±0.0135	0±0	0.0039±0.0078
k__Bacteria	g__Faecalibacterium	4.395E-01	7.046E-01	4.465E-02	0.3926±1.1405	0.0472±0.0309	0.0695±0.0316
k__Bacteria	g__FFCH10602	5.705E-01	6.850E-01	3.070E-02	0.0004±0.0018	0±0	0±0
k__Bacteria	g__Fibrobacter	4.475E-01	7.095E-01	4.369E-02	0.0078±0.0219	0±0	0.0039±0.011
k__Bacteria	g__Fimbriimonas	3.333E-01	7.086E-01	5.921E-02	0±0	0.0007±0.0023	0±0
k__Bacteria	g__Finegoldia	5.705E-01	7.687E-01	3.070E-02	0.0014±0.0057	0±0	0±0
k__Bacteria	g__Flavobacterium	3.158E-01	7.617E-01	6.202E-02	0.0016±0.0067	0.0008±0.0027	0.0102±0.0288
k__Bacteria	g__Flexispira	2.587E-01	7.426E-01	7.236E-02	2.8912±6.9696	0.2989±0.4032	0.1555±0.1262
k__Bacteria	g__Fluviicola	3.298E-01	7.634E-01	5.976E-02	0.0062±0.015	0±0	0.0034±0.0067
k__Bacteria	g__Fructobacillus	9.710E-02	6.635E-01	1.215E-01	0±0	0.0014±0.003	0±0
k__Bacteria	g__Fusobacterium	9.595E-02	7.443E-01	1.221E-01	2.5666±4.3773	0.0259±0.0453	0.8685±1.1739
k__Bacteria	g__Gardnerella	1.224E-04	1.171E-02	3.937E-01	0.0009±0.0025	0.0418±0.0425	0±0
k__Bacteria	g__Gemella	3.360E-01	6.516E-01	5.879E-02	0.001±0.0028	0.0052±0.0173	0.0074±0.0094
k__Bacteria	g__Gluconobacter	6.446E-01	7.312E-01	2.410E-02	0.0044±0.018	0±0	0.0044±0.0082
k__Bacteria	g__Gordonia	4.133E-01	6.856E-01	4.791E-02	0.002±0.0064	0±0	0±0
k__Bacteria	g__Granulicatella	9.864E-01	9.864E-01	7.595E-04	0.001±0.0029	0.0008±0.0028	0.0009±0.0026
k__Bacteria	g__Haemophilus	8.668E-01	8.917E-01	7.908E-03	0.0019±0.0035	0.0014±0.0045	0.0011±0.003
k__Bacteria	g__Halomonas	3.333E-01	7.359E-01	5.921E-02	0±0	0.0008±0.0026	0±0
k__Bacteria	g__Helcococcus	5.705E-01	6.766E-01	3.070E-02	0.0005±0.0019	0±0	0±0
k__Bacteria	g__Helicobacter	1.199E-04	1.721E-02	3.944E-01	0.0306±0.0712	0.7372±0.677	0.1128±0.1986
k__Bacteria	g__Holdemania	1.120E-01	6.696E-01	1.145E-01	0.006±0.0094	0±0	0.0026±0.0073
k__Bacteria	g__Hydrogenophaga	5.705E-01	7.759E-01	3.070E-02	0.0005±0.0021	0±0	0±0
k__Bacteria	g__Hyphomicrobium	1.151E-01	6.739E-01	1.132E-01	0.0004±0.0018	0±0	0.0022±0.0041
k__Bacteria	g__Iamia	1.916E-01	6.705E-01	8.772E-02	0±0	0±0	0.0011±0.0032
k__Bacteria	g__Janibacter	5.705E-01	7.987E-01	3.070E-02	0.0005±0.0021	0±0	0±0
k__Bacteria	g__Janthinobacterium	1.916E-01	7.049E-01	8.772E-02	0±0	0±0	0.0013±0.0037
k__Bacteria	g__Jeotgalicoccus	3.937E-01	6.766E-01	5.047E-02	0.0175±0.0453	0±0	0.0156±0.0296
k__Bacteria	g__Kaistobacter	4.694E-01	7.204E-01	4.115E-02	0.0037±0.0111	0±0	0.0031±0.0044
k__Bacteria	g__Kingella	5.705E-01	7.375E-01	3.070E-02	0.0004±0.0018	0±0	0±0
k__Bacteria	g__Klebsiella	9.621E-01	9.655E-01	2.142E-03	0.1473±0.342	0.161±0.1901	0.1835±0.3534
k__Bacteria	g__Kocuria	6.319E-01	7.196E-01	2.518E-02	0.0179±0.0397	0.0131±0.0201	0.0057±0.0128
k__Bacteria	g__Kurthia	5.705E-01	8.026E-01	3.070E-02	0.0005±0.0022	0±0	0±0
k__Bacteria	g__L7A_E11	3.333E-01	6.552E-01	5.921E-02	0±0	0.0007±0.0025	0±0
k__Bacteria	g__Labrys	2.301E-01	6.953E-01	7.837E-02	0.0004±0.0018	0.0024±0.0058	0±0
k__Bacteria	g__Laceyella	3.333E-01	7.592E-01	5.921E-02	0±0	0.0008±0.0025	0±0
k__Bacteria	g__Lachnobacterium	3.799E-01	6.648E-01	5.235E-02	0.7833±2.0977	0.0015±0.0034	1.2097±2.7669
k__Bacteria	g__Lachnospira	3.599E-02	4.304E-01	1.686E-01	1.0472±1.3784	0.0105±0.016	0.3551±0.8548
k__Bacteria	g__Lactobacillus	1.068E-01	6.966E-01	1.169E-01	0.021±0.0339	0.0694±0.1074	0.0147±0.018
k__Bacteria	g__Lactococcus	2.359E-02	3.762E-01	1.879E-01	0.0805±0.1278	0.0706±0.1477	0.3069±0.3391
k__Bacteria	g__Lautropia	2.329E-01	6.964E-01	7.775E-02	0.0024±0.005	0.0007±0.0024	0.0048±0.0074
k__Bacteria	g__Leptolyngbya	1.916E-01	7.430E-01	8.772E-02	0±0	0±0	0.0009±0.0027
k__Bacteria	g__Leptotrichia	1.203E-01	6.903E-01	1.110E-01	0.001±0.0027	0.0007±0.0025	0.0086±0.0185
k__Bacteria	g__Leucobacter	4.539E-01	7.157E-01	4.294E-02	0.0034±0.0085	0±0	0.0039±0.011
k__Bacteria	g__Leuconostoc	2.974E-01	7.688E-01	6.516E-02	0.2287±0.7638	0.0268±0.0453	0.6166±1.3121
k__Bacteria	g__Limnobacter	5.705E-01	7.545E-01	3.070E-02	0.0004±0.0018	0±0	0±0
k__Bacteria	g__Loktanella	5.705E-01	7.580E-01	3.070E-02	0.0004±0.0018	0±0	0±0
k__Bacteria	g__Luteimonas	7.064E-01	7.565E-01	1.912E-02	0.0027±0.0091	0.0008±0.0027	0.0033±0.0067
k__Bacteria	g__Lysinibacillus	5.705E-01	6.879E-01	3.070E-02	0.0032±0.0133	0±0	0±0
k__Bacteria	g__Lysobacter	5.705E-01	7.408E-01	3.070E-02	0.0005±0.0021	0±0	0±0
k__Bacteria	g__Mannheimia	5.705E-01	7.651E-01	3.070E-02	0.0005±0.0021	0±0	0±0
k__Bacteria	g__Megamonas	2.138E-01	6.741E-01	8.215E-02	2.2227±2.9382	4.3076±6.567	6.5004±8.1252
k__Bacteria	g__Megasphaera	1.226E-01	6.901E-01	1.101E-01	0.8727±1.1966	3.8889±4.8972	3.2609±5.7183
k__Bacteria	g__Mesorhizobium	6.470E-01	7.311E-01	2.390E-02	0.0009±0.0037	0±0	0.0011±0.0032
k__Archaea	g__Methanobrevibacter	5.274E-01	7.843E-01	3.492E-02	0.2415±0.8441	0±0	0.0635±0.0752
k__Archaea	g__Methanocalculus	5.705E-01	6.710E-01	3.070E-02	0.0005±0.0022	0±0	0±0
k__Archaea	g__Methanocorpusculum	1.622E-01	8.169E-01	9.610E-02	0.0102±0.0288	0±0	0.0522±0.1205
k__Archaea	g__Methanosphaera	2.856E-01	7.591E-01	6.724E-02	0.0505±0.1213	0.0014±0.0032	0.0123±0.0318
k__Bacteria	g__Methylibium	3.333E-01	7.247E-01	5.921E-02	0±0	0.0008±0.0026	0±0
k__Bacteria	g__Methylobacterium	1.016E-01	6.784E-01	1.193E-01	0±0	0.0033±0.0061	0.0013±0.0037
k__Bacteria	g__Methylotenera	3.145E-01	7.650E-01	6.224E-02	0.001±0.0028	0±0	0±0
k__Bacteria	g__Microbacterium	5.717E-01	6.697E-01	3.058E-02	0.0116±0.0246	0.0061±0.0114	0.0191±0.0406
k__Bacteria	g__Micrococcus	3.685E-01	6.529E-01	5.395E-02	0.0032±0.0098	0±0	0±0
k__Bacteria	g__Microlunatus	1.916E-01	7.234E-01	8.772E-02	0±0	0±0	0.0023±0.0064
k__Bacteria	g__Mitsuokella	7.698E-01	8.063E-01	1.443E-02	0.0005±0.0022	0.0015±0.0049	0.0013±0.0037
k__Bacteria	g__Mogibacterium	1.080E-01	6.738E-01	1.163E-01	0.0069±0.014	0±0	0±0
k__Bacteria	g__Moraxella	5.705E-01	7.118E-01	3.070E-02	0.001±0.0043	0±0	0±0
k__Bacteria	g__Morganella	2.174E-01	6.710E-01	8.127E-02	0.0596±0.1643	0.0107±0.0172	0.2736±0.6644
k__Bacteria	g__Mucispirillum	6.673E-01	7.394E-01	2.222E-02	0.0041±0.013	0.0007±0.0023	0.0026±0.0073
k__Bacteria	g__Mycetocola	5.705E-01	8.105E-01	3.070E-02	0.0005±0.0019	0±0	0±0
k__Bacteria	g__Mycobacterium	9.156E-01	9.352E-01	4.886E-03	0.0018±0.0043	0.0016±0.0037	0.0024±0.0045
k__Bacteria	g__Mycoplana	1.916E-01	8.206E-01	8.772E-02	0±0	0±0	0.0034±0.0096
k__Bacteria	g__Myroides	7.208E-01	7.661E-01	1.803E-02	0.0582±0.2352	0.0355±0.0982	0±0
k__Bacteria	g__Neisseria	2.411E-01	7.060E-01	7.599E-02	0.0039±0.0077	0.0085±0.0151	0.0218±0.0468
k__Bacteria	g__Nesterenkonia	4.293E-01	7.001E-01	4.589E-02	0.0145±0.0423	0±0	0.0143±0.0202
k__Bacteria	g__Nitrospira	5.705E-01	6.997E-01	3.070E-02	0.0022±0.009	0±0	0±0
k__Bacteria	g__Nocardia	6.644E-01	7.391E-01	2.246E-02	0.0025±0.0104	0±0	0.0011±0.0032
k__Bacteria	g__Nocardioides	1.916E-01	6.787E-01	8.772E-02	0±0	0±0	0.0019±0.0053
k__Bacteria	g__Nonomuraea	5.705E-01	7.909E-01	3.070E-02	0.0005±0.0021	0±0	0±0
k__Bacteria	g__Novosphingobium	3.231E-02	4.216E-01	1.736E-01	0±0	0.0038±0.0068	0±0
k__Bacteria	g__Ochrobactrum	4.036E-01	6.813E-01	4.916E-02	0.0041±0.0133	0±0	0±0
k__Bacteria	g__Odoribacter	1.849E-01	8.700E-01	8.951E-02	0.3917±0.8026	0.0052±0.0068	0.1253±0.1574
k__Bacteria	g__Oribacterium	1.916E-01	7.531E-01	8.772E-02	0±0	0±0	0.0012±0.0034
k__Bacteria	g__Oscillospira	1.594E-02	3.268E-01	2.054E-01	0.51±0.5526	0.0425±0.051	0.2795±0.2602
k__Bacteria	g__Oxalobacter	9.310E-02	7.422E-01	1.236E-01	0.0091±0.016	0±0	0.0019±0.0053
k__Bacteria	g__p-75-a5	1.916E-01	7.636E-01	8.772E-02	0±0	0±0	0.0011±0.0032
k__Bacteria	g__Paenibacillus	5.994E-01	6.909E-01	2.803E-02	0.005±0.0183	0.0014±0.0045	0±0
k__Bacteria	g__Paludibacter	7.932E-03	2.846E-01	2.356E-01	0±0	0.0007±0.0023	0.0105±0.0159
k__Bacteria	g__Parabacteroides	1.781E-01	8.666E-01	9.139E-02	4.2238±6.4113	0.9969±1.2716	2.151±1.7699
k__Bacteria	g__Paracoccus	1.029E-02	2.462E-01	2.245E-01	0.0035±0.0077	0±0	0.0114±0.0119
k__Bacteria	g__Paraprevotella	1.085E-01	6.627E-01	1.161E-01	0.392±0.5959	0.0053±0.0127	0.4049±0.6043
k__Bacteria	g__Parvimonas	3.333E-01	7.474E-01	5.921E-02	0±0	0.0007±0.0025	0±0
k__Bacteria	g__Pediococcus	1.882E-01	8.440E-01	8.861E-02	0.0137±0.0372	0±0	0.0507±0.1144
k__Bacteria	g__Pedobacter	1.916E-01	6.624E-01	8.772E-02	0±0	0±0	0.0011±0.0032
k__Bacteria	g__Peptococcus	6.634E-05	1.904E-02	4.140E-01	0.0214±0.0182	0.0809±0.0889	0.2065±0.1454
k__Bacteria	g__Peptoniphilus	5.322E-01	7.834E-01	3.443E-02	0.0009±0.0038	0±0	0.0019±0.0053
k__Bacteria	g__Peptostreptococcus	3.100E-01	7.669E-01	6.300E-02	0.0011±0.003	0±0	0±0
k__Bacteria	g__Phascolarctobacterium	2.745E-01	7.502E-01	6.931E-02	0.8932±2.1131	0.0114±0.0095	0.2721±0.4092
k__Bacteria	g__Phenylobacterium	4.691E-01	7.277E-01	4.118E-02	0.0004±0.0018	0±0	0.0011±0.0032
k__Bacteria	g__Phormidium	1.916E-01	7.743E-01	8.772E-02	0±0	0±0	0.0011±0.0032
k__Bacteria	g__Planctomyces	5.705E-01	7.510E-01	3.070E-02	0.0004±0.0018	0±0	0±0
k__Bacteria	g__Plesiomonas	5.705E-01	7.027E-01	3.070E-02	0.0005±0.0021	0±0	0±0
k__Bacteria	g__Porphyromonas	3.249E-01	7.643E-01	6.055E-02	0.0119±0.0316	0±0	0.0024±0.0067
k__Bacteria	g__Prevotella	3.956E-02	4.542E-01	1.643E-01	13.625±11.2428	13.8073±8.9363	24.3423±9.1821
k__Bacteria	g__Promicromonospora	3.333E-01	6.737E-01	5.921E-02	0±0	0.0007±0.0025	0±0
k__Bacteria	g__Propionibacterium	3.294E-01	7.687E-01	5.982E-02	0.0109±0.0303	0.0008±0.0026	0±0
k__Bacteria	g__Propionicimonas	1.916E-01	7.330E-01	8.772E-02	0±0	0±0	0.0011±0.0032
k__Bacteria	g__Proteus	1.962E-01	6.400E-01	8.649E-02	0.0041±0.0083	0.0023±0.0054	0.023±0.0546
k__Bacteria	g__Providencia	8.527E-01	8.803E-01	8.815E-03	0.0379±0.1199	0.0183±0.0412	0.0386±0.0989
k__Bacteria	g__Pseudomonas	3.700E-01	6.515E-01	5.374E-02	0.0141±0.0232	0.0064±0.0114	0.0045±0.0128
k__Bacteria	g__Pseudonocardia	6.383E-02	5.909E-01	1.418E-01	0.0018±0.005	0±0	0.0066±0.0104
k__Bacteria	g__Pseudoramibacter_Eubact	1.456E-02	3.214E-01	2.094E-01	0.0226±0.0308	0.0008±0.0027	0.0011±0.003
k__Bacteria	g__Pseudoxanthomonas	2.655E-01	7.543E-01	7.103E-02	0±0	0.0008±0.0027	0.0034±0.0096
k__Bacteria	g__Psychrobacter	9.701E-02	6.791E-01	1.216E-01	0±0	0.0015±0.0034	0±0
k__Bacteria	g__Rathayibacter	5.705E-01	6.822E-01	3.070E-02	0.0005±0.0019	0±0	0±0
k__Bacteria	g__RFN20	4.147E-02	4.578E-01	1.621E-01	1.1774±1.8972	1.6733±3.5057	4.3571±3.5752
k__Bacteria	g__Rhizobium	9.587E-01	9.689E-01	2.339E-03	0.001±0.0042	0.0008±0.0027	0.0013±0.0037
k__Bacteria	g__Rhodobacter	3.333E-01	6.932E-01	5.921E-02	0±0	0.0008±0.0026	0±0
k__Bacteria	g__Rhodococcus	4.891E-01	7.426E-01	3.896E-02	0.001±0.003	0.0015±0.0034	0±0
k__Bacteria	g__Rhodoplanes	3.096E-01	7.795E-01	6.306E-02	0.0026±0.0075	0±0	0±0
k__Bacteria	g__Rickettsiella	2.257E-01	6.892E-01	7.936E-02	0±0	0.0048±0.0135	0±0
k__Bacteria	g__Rikenella	3.099E-01	7.733E-01	6.302E-02	0.0009±0.0025	0±0	0±0
k__Bacteria	g__Roseburia	3.056E-01	7.832E-01	6.373E-02	2.4604±5.8732	0.0076±0.0092	1.1645±1.6835
k__Bacteria	g__Rothia	4.691E-01	7.238E-01	4.118E-02	0.0004±0.0018	0±0	0.0011±0.0032
k__Bacteria	g__Ruminobacter	3.110E-01	7.629E-01	6.282E-02	0.3901±1.5657	0.0023±0.004	1.6131±4.3225
k__Bacteria	g__Ruminococcus	1.825E-01	8.728E-01	9.018E-02	2.1028±4.3632	0.0343±0.0193	0.547±0.7572
k__Bacteria	g__Rummeliibacillus	5.338E-01	7.776E-01	3.428E-02	0.0915±0.3457	0.0016±0.0052	0.0043±0.0048
k__Bacteria	g__Saccharopolyspora	3.333E-01	6.785E-01	5.921E-02	0±0	0.0007±0.0023	0±0
k__Bacteria	g__Salinicoccus	3.489E-01	6.544E-01	5.683E-02	0.0022±0.0052	0±0	0.0012±0.0034
k__Bacteria	g__Salmonella	4.140E-01	6.828E-01	4.782E-02	0.0105±0.0295	0.0007±0.0025	0.0024±0.0067
k__Bacteria	g__Sarcina	6.537E-01	7.329E-01	2.334E-02	2.4346±9.4706	0.019±0.0402	1.6448±4.1186
k__Bacteria	g__Sedimentibacter	5.705E-01	6.794E-01	3.070E-02	0.0009±0.0038	0±0	0±0
k__Bacteria	g__Sediminicola	5.705E-01	8.227E-01	3.070E-02	0.0005±0.0019	0±0	0±0
k__Bacteria	g__Serratia	9.607E-02	7.256E-01	1.220E-01	0.0904±0.2411	0.0391±0.0815	2.5691±5.8699
k__Bacteria	g__Sharpea	4.778E-03	2.285E-01	2.569E-01	0±0	0.0031±0.0044	0±0
k__Bacteria	g__Slackia	1.890E-01	8.347E-01	8.839E-02	0.0351±0.064	0.0007±0.0025	0.0271±0.0424
k__Bacteria	g__SMB53	6.835E-01	7.487E-01	2.092E-02	0.0026±0.0108	0±0	0.0023±0.0064
k__Bacteria	g__Sodalis	1.725E-01	8.534E-01	9.303E-02	0±0	0.0036±0.0094	0±0
k__Bacteria	g__Solibacillus	3.620E-01	6.453E-01	5.488E-02	0.0123±0.0338	0±0	0.0157±0.0268
k__Bacteria	g__Sorangium	5.705E-01	8.269E-01	3.070E-02	0.0004±0.0018	0±0	0±0
k__Bacteria	g__Sphaerochaeta	8.826E-01	9.047E-01	6.911E-03	1.8114±2.3174	2.4594±5.1022	1.9208±2.3035
k__Bacteria	g__Sphingobacterium	3.199E-01	7.650E-01	6.136E-02	0.0213±0.0537	0.0039±0.006	0.0011±0.003
k__Bacteria	g__Sphingobium	2.967E-01	7.740E-01	6.528E-02	0.0011±0.0045	0.0031±0.0059	0±0
k__Bacteria	g__Sphingomonas	7.452E-01	7.863E-01	1.621E-02	0.0039±0.0089	0.0069±0.0136	0.0077±0.0186
k__Bacteria	g__Sphingopyxis	3.963E-01	6.770E-01	5.012E-02	0.001±0.0027	0±0	0.0023±0.0064
k__Bacteria	g__Spirosoma	1.916E-01	7.968E-01	8.772E-02	0±0	0±0	0.0032±0.0089
k__Bacteria	g__Staphylococcus	4.776E-01	7.291E-01	4.022E-02	0.0997±0.3356	0.0062±0.0121	0.0058±0.0086
k__Bacteria	g__Stenotrophomonas	4.557E-01	7.147E-01	4.272E-02	0.0228±0.0724	0.0034±0.0113	0±0
k__Bacteria	g__Steroidobacter	1.916E-01	8.085E-01	8.772E-02	0±0	0±0	0.0011±0.0032
k__Bacteria	g__Streptobacillus	5.705E-01	7.150E-01	3.070E-02	0.0005±0.0022	0±0	0±0
k__Bacteria	g__Streptococcus	3.208E-01	7.610E-01	6.120E-02	0.1211±0.2393	0.0308±0.0398	0.0463±0.0347
k__Bacteria	g__Streptomyces	4.199E-01	6.886E-01	4.707E-02	0.0035±0.0095	0±0	0.0022±0.0041
k__Bacteria	g__Succiniclasticum	1.916E-01	6.468E-01	8.772E-02	0±0	0±0	0.0012±0.0034
k__Bacteria	g__Succinivibrio	7.659E-02	6.280E-01	1.330E-01	3.8958±7.0289	0.0015±0.0033	0.3423±0.4893
k__Bacteria	g__Sutterella	3.551E-02	4.431E-01	1.693E-01	1.1049±0.8889	0.3458±0.5223	1.0115±0.6969
k__Bacteria	g__Synergistes	4.934E-02	4.883E-01	1.539E-01	0.0866±0.1442	0±0	0.0066±0.0081
k__Bacteria	g__Syntrophomonas	2.025E-01	6.458E-01	8.490E-02	0.002±0.0049	0±0	0±0
k__Bacteria	g__Tannerella	4.581E-01	7.146E-01	4.244E-02	0.0004±0.0018	0±0	0.0011±0.0032
k__Bacteria	g__Tepidimicrobium	5.705E-01	6.738E-01	3.070E-02	0.0005±0.0022	0±0	0±0
k__Bacteria	g__Terracoccus	3.333E-01	7.193E-01	5.921E-02	0±0	0.0007±0.0023	0±0
k__Bacteria	g__Thauera	5.705E-01	7.871E-01	3.070E-02	0.0005±0.002	0±0	0±0
k__Bacteria	g__Thiomonas	1.916E-01	7.140E-01	8.772E-02	0±0	0±0	0.0011±0.0032
k__Bacteria	g__Tindallia_Anoxynatronum	3.333E-01	6.833E-01	5.921E-02	0±0	0.0008±0.0027	0±0
k__Bacteria	g__Tolumonas	5.705E-01	7.834E-01	3.070E-02	0.0005±0.0021	0±0	0±0
k__Bacteria	g__Trabulsiella	9.584E-01	9.719E-01	2.358E-03	0.0114±0.0223	0.0092±0.0187	0.0101±0.0167
k__Bacteria	g__Treponema	1.599E-01	8.194E-01	9.683E-02	0.2158±0.4298	0.0212±0.0539	0.0247±0.0328
k__Bacteria	g__Trichococcus	4.056E-01	6.807E-01	4.890E-02	0.008±0.0159	0.0057±0.0098	0.0009±0.0026
k__Bacteria	g__Truepera	4.810E-02	4.930E-01	1.551E-01	0.0004±0.0018	0±0	0.0051±0.0096
k__Bacteria	g__Trueperella	5.705E-01	6.938E-01	3.070E-02	0.0004±0.0018	0±0	0±0
k__Bacteria	g__Turicibacter	5.749E-02	5.499E-01	1.467E-01	0.0024±0.0047	0.0014±0.0045	0.0079±0.0095
k__Archaea	g__vadinCA11	1.869E-01	8.516E-01	8.896E-02	0.1324±0.2777	0±0	0.041±0.0334
k__Bacteria	g__Vagococcus	8.125E-01	8.418E-01	1.147E-02	0.0016±0.0067	0.0016±0.0052	0.0034±0.0096
k__Bacteria	g__Variovorax	5.705E-01	6.908E-01	3.070E-02	0.0004±0.0018	0±0	0±0
k__Bacteria	g__Veillonella	2.704E-01	7.609E-01	7.007E-02	0.0054±0.0097	0.0015±0.0033	0.0099±0.0184
k__Bacteria	g__Vibrio	1.290E-01	7.122E-01	1.075E-01	0±0	0.0023±0.0055	0±0
k__Bacteria	g__Viridibacillus	5.705E-01	6.967E-01	3.070E-02	0.0005±0.0022	0±0	0±0
k__Bacteria	g__Wautersiella	7.038E-01	7.623E-01	1.932E-02	0.0332±0.1263	0.0293±0.0761	0±0
k__Bacteria	g__Weissella	3.508E-01	6.538E-01	5.653E-02	0.0015±0.0033	0±0	0.0009±0.0026
k__Bacteria	g__Williamsia	5.299E-01	7.840E-01	3.466E-02	0.0005±0.0021	0±0	0.0011±0.003
k__Bacteria	g__Wohlfahrtiimonas	3.333E-01	6.597E-01	5.921E-02	0±0	0.0023±0.0077	0±0
k__Bacteria	g__Xanthomonas	3.333E-01	7.653E-01	5.921E-02	0±0	0.0008±0.0025	0±0
k__Bacteria	g__Xylanimicrobium	4.388E-01	7.074E-01	4.473E-02	0.0016±0.0067	0±0	0.0032±0.0064
k__Bacteria	g__Xylella	3.333E-01	7.416E-01	5.921E-02	0±0	0.0007±0.0023	0±0
k__Bacteria	g__Yaniella	5.705E-01	7.277E-01	3.070E-02	0.0004±0.0018	0±0	0±0
k__Bacteria	g__YRC22	3.580E-01	6.463E-01	5.546E-02	0.021±0.0596	0.0023±0.004	0±0
k__Bacteria	g__za29	4.985E-01	7.531E-01	3.793E-02	0.001±0.0029	0±0	0.0013±0.0037

Table S2. Phylum-level analysis of slow loris microbiota. Correcting p-values have undergone Benjamini-Hochberg Correction.

Level_1	Level_2	p-values	p-values (corrected)	Effect size	Traditional Diet: mean rel. freq. (%) ± std. dev. (%)	Wild Diet: mean rel. freq. (%) ± std. dev. (%)	Improved Diet: mean rel. freq. (%) ± std. dev. (%)
k__Bacteria	p__Actinobacteria	0.0030	0.0990	0.2758	26.2779 ± 24.217	56.5585 ± 22.9703	27.0598 ± 17.5722
k__Bacteria	p__Firmicutes	0.0100	0.1645	0.2083	27.7031 ± 16.1564	14.5675 ± 9.0201	29.9708 ± 7.7243
k__Bacteria	p__TM7	0.0149	0.1644	0.2259	0.529 ± 1.9376	5.3223 ± 6.1541	1.7601 ± 2.4959
k__Bacteria	p__Synergistetes	0.0346	0.2858	0.1655	0.0704 ± 0.1125	0 ± 0	0.005 ± 0.0061
k__Bacteria	p__Bacteroidetes	0.0385	0.2542	0.1704	30.5975 ± 15.693	17.2295 ± 11.1346	27.3719 ± 7.4156
k__Bacteria	p__Tenericutes	0.0851	0.4680	0.1184	0.0435 ± 0.1344	0.0031 ± 0.0037	0.8761 ± 2.0248
k__Bacteria	p__Cyanobacteria	0.0870	0.4100	0.1213	0.4399 ± 0.9089	0.0226 ± 0.029	1.7779 ± 3.4602
k__Bacteria	p__[Thermi]	0.0975	0.4021	0.1269	0.0008 ± 0.0023	0 ± 0	0.0041 ± 0.0079
k__Bacteria	p__Lentisphaerae	0.1035	0.3795	0.1059	0.1943 ± 0.3431	0.0019 ± 0.0045	0.0889 ± 0.1407
k__Bacteria	p__TM6	0.1334	0.4403	0.0877	0 ± 0	0 ± 0	0.0008 ± 0.0023
k__Bacteria	p__Chloroflexi	0.1553	0.4659	0.0831	0.0091 ± 0.0236	0.0019 ± 0.0033	0.0174 ± 0.0199
k__Bacteria	p__Proteobacteria	0.1848	0.5082	0.0983	9.7394 ± 8.9195	4.1636 ± 2.5509	8.7853 ± 8.4777
k__Bacteria	p__Fusobacteria	0.1916	0.4863	0.1279	2.1549 ± 3.6552	0.0225 ± 0.0366	0.6013 ± 0.8046
k__Bacteria	p__Chlamydiae	0.1916	0.4515	0.0877	0 ± 0	0 ± 0	0.0008 ± 0.0023
k__Bacteria	p__Elusimicrobia	0.2098	0.4615	0.0895	0.0149 ± 0.0404	0 ± 0	0.1272 ± 0.3285
k__Archaea	p__Euryarchaeota	0.2535	0.5229	0.0734	0.3784 ± 0.8736	0.0013 ± 0.0028	0.1259 ± 0.1001
k__Bacteria	p__Armatimonadetes	0.2988	0.5800	0.0622	0 ± 0	0.0006 ± 0.0021	0.0017 ± 0.0047
k__Bacteria	p__Planctomycetes	0.3149	0.5773	0.0592	0.0062 ± 0.0163	0 ± 0	0.0017 ± 0.0031
k__Bacteria	p__Verrucomicrobia	0.3336	0.5793	0.0649	0.1526 ± 0.3318	0.0093 ± 0.0181	0.083 ± 0.1328
k__Bacteria	p__GN01	0.4312	0.7114	0.0307	0.0004 ± 0.0017	0 ± 0	0 ± 0
k__Bacteria	p__OD1	0.5163	0.8113	0.0307	0.0008 ± 0.0034	0 ± 0	0 ± 0
k__Bacteria	p__WPS-2	0.5368	0.8052	0.0307	0.0012 ± 0.0051	0 ± 0	0 ± 0
k__Bacteria	p__Deferribacteres	0.5705	0.6972	0.0245	0.0037 ± 0.0122	0.0006 ± 0.0021	0.0017 ± 0.0047
k__Archaea	p__Crenarchaeota	0.5705	0.8185	0.0307	0.0004 ± 0.0017	0 ± 0	0 ± 0
k__Bacteria	p__Fibrobacteres	0.5705	0.7844	0.0457	0.0062 ± 0.0175	0 ± 0	0.0025 ± 0.0071
k__Bacteria	p__OP3	0.5705	0.7530	0.0307	0.0029 ± 0.0119	0 ± 0	0 ± 0
k__Bacteria	p__BRC1	0.5705	0.7241	0.0307	0.0004 ± 0.0017	0 ± 0	0 ± 0
k__Bacteria	p__Gemmatimonadetes	0.5705	0.6723	0.0171	0.0029 ± 0.0119	0.0006 ± 0.0021	0.0008 ± 0.0023
k__Bacteria	p__Nitrospirae	0.5705	0.6492	0.0307	0.0021 ± 0.0085	0 ± 0	0 ± 0
k__Bacteria	p__Chlorobi	0.5705	0.6275	0.0340	0.0004 ± 0.0017	0 ± 0	0.0008 ± 0.0024
k__Bacteria	p__Acidobacteria	0.6398	0.6811	0.0361	0.007 ± 0.0238	0.0006 ± 0.0021	0.0008 ± 0.0024
k__Bacteria	p__GAL15	0.7327	0.7556	0.0307	0.0008 ± 0.0034	0 ± 0	0 ± 0
k__Bacteria	p__Spirochaetes	0.8401	0.8401	0.0096	1.6587 ± 2.0309	2.0936 ± 4.2983	1.3337 ± 1.538

Table S3. Differences between treatment groups in predicted metabolic pathways

Level_1	Level_2	p-values	p-values (corrected)	Effect size	Traditional Diet: mean rel. freq. (%)	Traditional Diet: std. dev. (%)	Wild Diet: mean rel. freq. (%)	Wild Diet: std. dev. (%)	Improved Diet: mean rel. freq. (%)	Improved Diet: std. dev. (%)
Organismal Systems	Immune System	0.00000004	0.00000244	0.61286594	0.0814	0.0160	0.0353	0.0215	0.0768	0.0066
Metabolism	Energy metabolism	0.00000028	0.00000894	0.56759506	0.8314	0.1145	0.5624	0.1078	0.8028	0.0777
Human Diseases	Cancers	0.00000309	0.00006586	0.50584437	0.1043	0.0270	0.0483	0.0277	0.1032	0.0175
Metabolism	Lipid metabolism	0.00000536	0.00006858	0.49047258	0.1152	0.0255	0.0599	0.0308	0.1127	0.0166
Organismal Systems	Environmental Adaptation	0.00000477	0.00007636	0.49373991	0.1605	0.0241	0.1114	0.0255	0.1604	0.0158
Poorly Characterized	General function prediction only	0.00000773	0.00008249	0.47997869	3.4719	0.1579	3.1553	0.1768	3.4842	0.1068
Organismal Systems	Endocrine System	0.00014632	0.00133779	0.38770399	0.3247	0.0526	0.4034	0.0701	0.2881	0.0371
Metabolism	Nucleotide metabolism	0.00034861	0.00278890	0.35744845	0.0391	0.0129	0.0590	0.0055	0.0602	0.0210
Metabolism	Amino Acid Metabolism	0.00100370	0.00642371	0.31856798	9.8911	0.3665	10.5495	0.5260	9.7783	0.5807
Cellular Processes and Signaling	Germination	0.00099492	0.00707498	0.31890072	0.0206	0.0144	0.0022	0.0026	0.0159	0.0130
Metabolism	Biosynthesis and biodegradation of secondary metabolites	0.00137651	0.00734141	0.30650493	0.0577	0.0176	0.0270	0.0224	0.0529	0.0227
Cellular Processes and Signaling	Other transporters	0.00156904	0.00772453	0.30144283	0.2432	0.0382	0.1900	0.0502	0.2541	0.0287
Environmental Information Processing	Signaling Molecules and Interaction	0.00134327	0.00781537	0.30744628	0.2094	0.0416	0.2635	0.0427	0.1989	0.0343
Organismal Systems	Excretory System	0.00175331	0.00801515	0.29712016	0.0177	0.0092	0.0049	0.0070	0.0122	0.0087
Cellular Processes and Signaling	Sporulation	0.00221627	0.00945610	0.28791042	0.4922	0.2880	0.1542	0.0792	0.4564	0.2656
Metabolism	Nucleotide Metabolism	0.00308649	0.01234595	0.27468641	4.1942	0.1738	4.5341	0.3388	4.1817	0.2745
Cellular Processes	Transport and Catabolism	0.00342728	0.01290269	0.27045391	0.2710	0.0670	0.3125	0.0607	0.2136	0.0345
Metabolism	Xenobiotics Biodegradation and Metabolism	0.00454446	0.01615809	0.25892845	1.6835	0.1596	1.8855	0.1786	1.6987	0.0968
Environmental Information Processing	Signal Transduction	0.00537744	0.01811349	0.25196680	1.4822	0.2074	1.1774	0.2440	1.5003	0.3164
Genetic Information Processing	Replication, recombination and repair proteins	0.00668948	0.02140634	0.24283859	0.7853	0.0768	0.8915	0.0660	0.8721	0.1234
Cellular Processes and Signaling	Membrane and intracellular structural molecules	0.00714062	0.02176188	0.24008836	0.4739	0.1321	0.3164	0.1330	0.4591	0.0999
Metabolism	Glycan biosynthesis and metabolism	0.00778491	0.02264702	0.23643250	0.0489	0.0253	0.0227	0.0141	0.0476	0.0209
Metabolism	Carbohydrate metabolism	0.00841180	0.02340675	0.23314006	0.1602	0.0357	0.1902	0.0399	0.1382	0.0251
Genetic Information Processing	Translation	0.01081216	0.02883243	0.22237015	5.9694	0.3843	6.4472	0.5430	5.8271	0.4993
Cellular Processes and Signaling	Electron transfer carriers	0.01522865	0.03898535	0.20743161	0.0321	0.0183	0.0120	0.0133	0.0328	0.0232
Metabolism	Metabolism of Other Amino Acids	0.01875238	0.04615970	0.19821360	1.5280	0.0857	1.6128	0.0571	1.5533	0.0669
Environmental Information Processing	Membrane Transport	0.01983620	0.04701915	0.19570686	12.5168	1.2713	13.7421	0.8603	13.1637	0.8915
Genetic Information Processing	Folding, Sorting and Degradation	0.03054798	0.06306680	0.17617994	2.4822	0.1569	2.3395	0.1168	2.4031	0.1026
Metabolism	Energy Metabolism	0.02963801	0.06322776	0.17756284	5.6084	0.3353	5.2733	0.3306	5.4049	0.2507
Human Diseases	Metabolic Diseases	0.02905683	0.06412541	0.17846722	0.1077	0.0097	0.1211	0.0090	0.1159	0.0199
Metabolism	Amino acid metabolism	0.02867670	0.06554673	0.17906802	0.1727	0.0458	0.1513	0.0265	0.2000	0.0328
Cellular Processes and Signaling	Other ion-coupled transporters	0.05214967	0.10429935	0.15133518	1.1926	0.1507	1.1170	0.1275	1.2942	0.1833
Genetic Information Processing	Protein folding and associated processing	0.06324624	0.11565027	0.14219057	0.6919	0.0890	0.7595	0.0578	0.6893	0.0761
Cellular Processes	Cell Motility	0.06037603	0.11709291	0.14440103	2.1516	0.9122	1.4905	0.5337	2.2236	0.7371
Genetic Information Processing	Translation proteins	0.06309402	0.11876521	0.14230540	0.9152	0.0376	0.9577	0.0566	0.9381	0.0423
Organismal Systems	Nervous System	0.07042124	0.12180971	0.13705417	0.1040	0.0109	0.1050	0.0123	0.0926	0.0156
Genetic Information Processing	Transcription	0.06895517	0.12258698	0.13806218	2.6300	0.3283	2.3957	0.1959	2.6518	0.2675
Metabolism	Glycan Biosynthesis and Metabolism	0.07570879	0.12750954	0.13357625	2.2443	0.4247	1.9239	0.2855	2.1807	0.2900
Genetic Information Processing	Replication and Repair	0.08075719	0.13252461	0.13046345	8.9569	0.3175	9.3210	0.5667	8.9220	0.4940
Genetic Information Processing	Restriction enzyme	0.08392739	0.13428382	0.12860137	0.1864	0.0317	0.1605	0.0300	0.1826	0.0253
Metabolism	Metabolism of cofactors and vitamins	0.09988814	0.15592295	0.12013217	0.1195	0.0308	0.1013	0.0224	0.1255	0.0189
Cellular Processes and Signaling	Signal transduction mechanisms	0.10940071	0.16670584	0.11567433	0.4307	0.0409	0.4289	0.0455	0.4670	0.0444
Metabolism	Biosynthesis of Other Secondary Metabolites	0.11973695	0.17821313	0.11122781	0.9282	0.0952	0.9675	0.0451	0.8929	0.0711
Metabolism	Lipid Metabolism	0.12764618	0.18566717	0.10806385	2.7024	0.1937	2.5557	0.1808	2.6236	0.1614
Human Diseases	Neurodegenerative Diseases	0.17445584	0.24811497	0.09244821	0.1489	0.1357	0.0880	0.0226	0.0903	0.0210
Cellular Processes	Cell Communication	0.19156351	0.25541801	0.08771930	0.0000	0.0000	0.0000	0.0000	0.0000	0.0001
Organismal Systems	Circulatory System	0.18709410	0.26030484	0.08891500	0.0145	0.0278	0.0033	0.0034	0.0017	0.0009
Organismal Systems	Sensory System	0.19156351	0.26085244	0.08771930	0.0000	0.0000	0.0000	0.0000	0.0000	0.0000
Cellular Processes	Cell Growth and Death	0.22440017	0.29309410	0.07966544	0.5072	0.0318	0.5379	0.0507	0.5184	0.0569
Cellular Processes and Signaling	Pores ion channels	0.25252556	0.31689482	0.07360812	0.3126	0.0994	0.2605	0.0812	0.3197	0.0793
Metabolism	Metabolism of Cofactors and Vitamins	0.25210606	0.32269576	0.07369368	4.3093	0.2157	4.4167	0.3292	4.5074	0.3338
Human Diseases	Infectious Diseases	0.52772715	0.64951033	0.03488670	0.3820	0.0294	0.3897	0.0308	0.3987	0.0480
Human Diseases	Cardiovascular Diseases	0.66845247	0.79223996	0.02212871	0.0003	0.0004	0.0002	0.0003	0.0003	0.0004
Metabolism	Carbohydrate Metabolism	0.66241957	0.79990288	0.02262111	10.9180	0.7728	10.8193	0.3304	10.6979	0.2846
Cellular Processes and Signaling	Cell motility and secretion	0.71178766	0.81347161	0.01871028	0.1795	0.0478	0.1711	0.0433	0.1900	0.0609
Metabolism	Others	0.72597520	0.81513005	0.01763375	0.9069	0.0646	0.9103	0.1186	0.8789	0.1020
Metabolism	Enzyme Families	0.71063058	0.82691558	0.01879897	2.1338	0.0884	2.1125	0.1657	2.0832	0.1941
Human Diseases	Immune System Diseases	0.76484251	0.84396415	0.01478327	0.0497	0.0094	0.0507	0.0087	0.0475	0.0111
Cellular Processes and Signaling	Cell division	0.79717608	0.85032115	0.01251435	0.0655	0.0078	0.0640	0.0074	0.0661	0.0068
Genetic Information Processing	Transcription related proteins	0.79322582	0.86044834	0.01278684	0.0059	0.0051	0.0062	0.0035	0.0073	0.0062
Organismal Systems	Digestive System	0.93447986	0.98043788	0.00375766	0.0484	0.0137	0.0488	0.0072	0.0469	0.0133
Cellular Processes and Signaling	Inorganic ion transport and metabolism	0.95565152	0.98647899	0.00251694	0.2040	0.0343	0.2081	0.0407	0.2034	0.0491
Metabolism	Metabolism of Terpenoids and Polyketides	0.99317015	0.99317015	0.00038067	1.6574	0.0644	1.6546	0.0533	1.6554	0.0843
Poorly Characterized	Function unknown	0.98002907	0.99558509	0.00112010	1.3259	0.1538	1.3181	0.1438	1.3340	0.2348
